# Reconstitution of Microtubule Nucleation *In Vitro* Reveals Novel Roles for Mzt1

**DOI:** 10.1016/j.cub.2023.04.030

**Published:** 2023-05-08

**Authors:** Su Ling Leong, Eric M. Lynch, Juan Zou, Ye Dee Tay, Weronika E. Borek, Maarten W. Tuijtel, Juri Rappsilber, Kenneth E. Sawin

(Current Biology *29*, 2199–2207.e1–e10; July 8, 2019)

In the original publication of this paper, James Le Cornu, who contributed to the electron microscopy negative staining and data collection found in Figures 2D and S2F, was not included in the original list of authors due to an oversight in image file organization in which images from multiple negatively stained grids were combined in a single folder. In addition, for the images shown in Figures 2D and S2F, the MGM concentration used was 10-fold higher than originally described, and there were minor differences in the staining and washing steps. All these differences have since been corrected online. All co-authors on the manuscript have approved these corrections. The authors apologize for the error.

