# Supplementary material for: Reconstitution of Microtubule Nucleation In Vitro Reveals Novel Roles for Mzt1
Source: Curr Biol. 2019 Jul 8;29(13):2199–2207.e10. doi: 10.1016/j.cub.2019.05.058 (PMC6616311; doi:10.1016/j.cub.2019.05.058)
Supplement: Document S1. Figures S1–S4 and Table S1 [file mmc1.pdf]

**Current Biology, Volume *29***

## Supplemental Information

# Reconstitution of Microtubule Nucleation *In Vitro* Reveals Novel Roles for Mzt1

**Su Ling Leong, Eric M. Lynch, Juan Zou, Ye Dee Tay, Weronika E. Borek, Maarten W. Tuijtel, Juri Rappsilber, and Kenneth E. Sawin**

A

| | *S. pombe* | *S. cerevisiae* | *C. albicans* | *H. sapiens* | *D. melanogaster* |
|---|---|---|---|---|---|
| γ-TuSC proteins | Gtb1 | Tub4 | *Ca*Tub4 | γ-tubulin | γ-tubulin |
| | Alp4 | Spc97 | *Ca*Spc97 | GCP2 | Grip84 |
| | Alp6 | Spc98 | *Ca*Spc98 | GCP3 | Grip91 |
| γ-TuRC-specific proteins | Gfh1 | (none) | (none) | GCP4 | Grip75 |
| | Mod21 | (none) | (none) | GCP5 | Grip128 |
| | Alp16 | (none) | (none) | GCP6 | Grip163 |
| Mzt1 | Mzt1 | (none) | *Ca*Mzt1 | MOZART1 | Mzt1 |
| CM1 proteins | Pcp1<br>Mto1 | Spc110<br>Spc72 | *Ca*Spc110<br>*Ca*Spc72 | CDK5RAP2<br>myomegalin | Cnn |
| (Mto1 interactor & possible multimerizer) | (Mto2) | ? | ? | ? | ? |

B

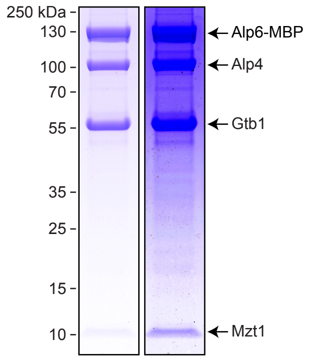


C

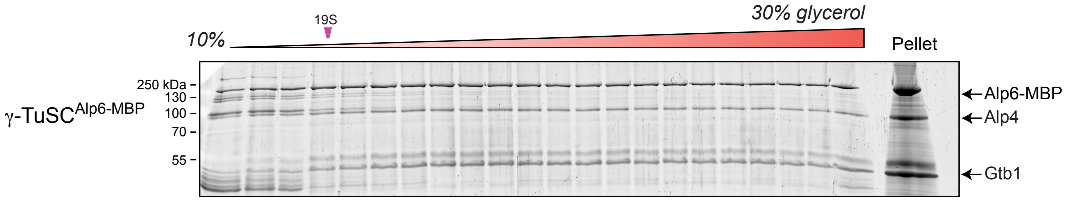


D

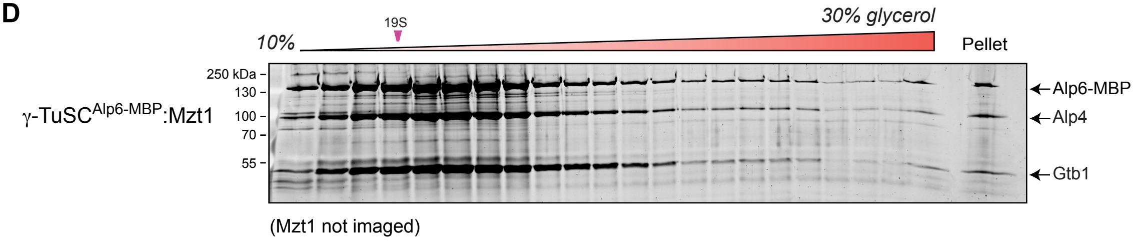

**Figure S1. Further characterization of γ-TuSC$^{Alp6\text{-}MBP}$ and γ-TuSC$^{Alp6\text{-}MBP}$:Mzt1. Related to Figure 1.**

**(A)** Nomenclature for the conserved γ-tubulin complex proteins and regulators relevant to this study. **(B)** SDS-PAGE of γ-TuSC$^{Alp6\text{-}MBP}$:Mzt1, shown at the two different contrast levels used for the composite in Figure 1D. **(C)** SDS-PAGE of 45-minute density-gradient centrifugation of γ-TuSC$^{Alp6\text{-}MBP}$ (SYPRO Ruby stain). Pelleting of γ-TuSC$^{Alp6\text{-}MBP}$ under these conditions indicates a sedimentation coefficient of at least 150S. See also Figure 1C. **(D)** SDS-PAGE of 45-minute density-gradient centrifugation of γ-TuSC$^{Alp6\text{-}MBP}$:Mzt1 (SYPRO Ruby stain). Because of the amount of protein loaded, Mzt1 was not visualized in this experiment. See also Figure 1F.

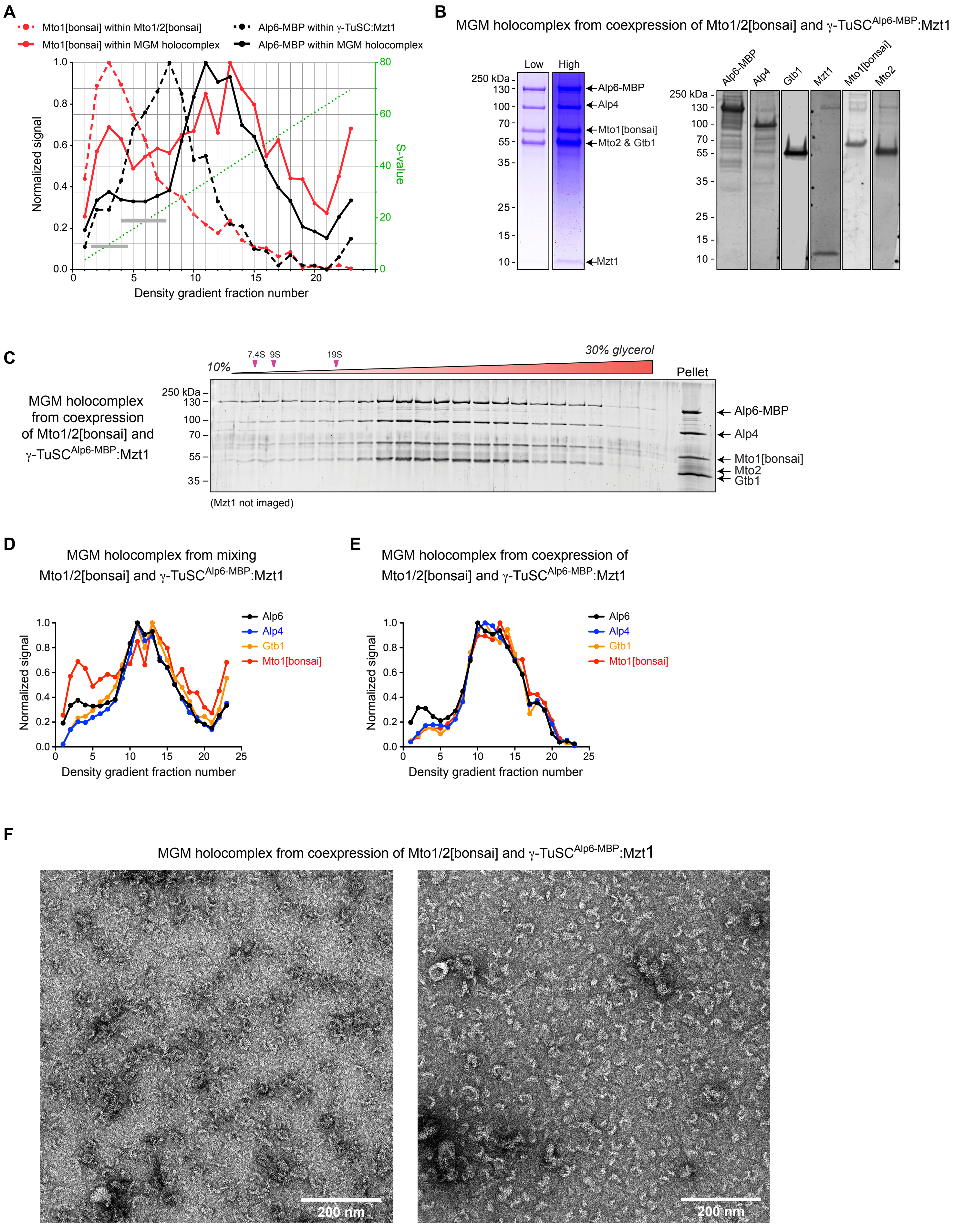
A
Mto1[bonsai] within Mto1/2[bonsai]
Mto1[bonsai] within MGM holocomplex
Alp6-MBP within γ-TuSC:Mzt1
Alp6-MBP within MGM holocomplex
Normalized signal
S-value
Density gradient fraction number
B
MGM holocomplex from coexpression of Mto1/2[bonsai] and γ-TuSC^Alp6-MBP:Mzt1
Low
High
250 kDa
Alp6-MBP
Alp4
Mto1[bonsai]
Mto2 & Gtb1
Mzt1
Gtb1
Mto2
C
10%
30% glycerol
7.4S
9S
19S
Pellet
MGM holocomplex from coexpression of Mto1/2[bonsai] and γ-TuSC^Alp6-MBP:Mzt1
(Mzt1 not imaged)
D
MGM holocomplex from mixing Mto1/2[bonsai] and γ-TuSC^Alp6-MBP:Mzt1
Alp6
Alp4
Gtb1
Mto1[bonsai]
E
MGM holocomplex from coexpression of Mto1/2[bonsai] and γ-TuSC^Alp6-MBP:Mzt1
F
MGM holocomplex from coexpression of Mto1/2[bonsai] and γ-TuSC^Alp6-MBP:Mzt1
200 nm
200 nm

**Figure S2. Further characterization of MGM holocomplex. Related to Figure 2.**
**(A)** Comparison of density-gradient sedimentation profiles of Mto1[bonsai] when present in Mto1/2[bonsai] complex (dashed red line; quantification of Figure 2A) vs. when present in MGM holocomplex generated by mixing Mto1/2[bonsai] with γ-TuSC$^{Alp6\text{-}MBP}$:Mzt1 (solid red line; quantification of Figure 2B), and of Alp6-MBP when present in γ-TuSC$^{Alp6\text{-}MBP}$:Mzt1 (dashed black line; quantification of Figure 1F) vs. when present in MGM holocomplex generated by mixing (solid black line; quantification of Figure 2B). Signals for a given protein were normalized to the highest signal for that protein in the gradient. Upon mixing to generate the MGM holocomplex, Mto1[bonsai] and Alp6-MBP (representative of Mto1/2[bonsai] and γ-TuSC$^{Alp6\text{-}MBP}$:Mzt1 respectively) cosediment, with significant sedimentation shifts compared to when they are in separate complexes. Gray bars indicate range of full-width half-maximal sedimentation of protein standards β-amylase (9S) and thyroglobulin (19S). Dashed green line shows relationship between fraction number and estimated S-value (right-hand Y-axis), based on isokinetic sedimentation. **(B)** SDS-PAGE (Coomassie Blue stain) and western blotting of MGM holocomplex purified from coexpression of Mto1/2[bonsai] with γ-TuSC$^{Alp6\text{-}MBP}$:Mzt1 (amylose purification). Gel stain is shown at two different contrast levels, to visualize both high and low molecular-weight proteins. Western blotting confirms identity of different proteins. Note comigration of Mto2 and γ-tubulin (Gtb1) on this gel system. **(C)** SDS-PAGE of 80-minute density-gradient centrifugation of MGM purified from coexpression of Mto1/2[bonsai] and γ-TuSC$^{Alp6\text{-}MBP}$:Mzt1 (SYPRO Ruby stain). Sedimentation of MGM purified from coexpression is essentially identical to that of MGM generated by combining independent complexes (Figure 2B). Because of the amount of protein loaded, Mzt1 was not visualized in this experiment. **(D, E)** Density-gradient sedimentation profiles of proteins in MGM generated by mixing Mto1/2[bonsai] and γ-TuSC$^{Alp6\text{-}MBP}$:Mzt1 (D) and in MGM purified from coexpression of Mto1/2[bonsai] and γ-TuSC$^{Alp6\text{-}MBP}$:Mzt1 (E). Data are from quantification of SYPRO Ruby staining shown in Figures 2B and S2C. Signals were normalized as in A. Very similar profiles would suggest that relative stoichiometry of MGM proteins does not change through the gradient. In D, the relative stoichiometry of MGM proteins is mostly constant throughout the peak. An excess of Mto1[bonsai] in the first few fractions may be the result of mixing γ-TuSC$^{Alp6\text{-}MBP}$:Mzt1 with a slight excess of Mto1/2[bonsai] (which itself peaks in fraction 3, as shown in A). A small excess of Alp6 in the first few fractions is likely due to fact that the MBP tag on Alp6 was used for purification of γ-TuSC$^{Alp6\text{-}MBP}$:Mzt1; as a result, purified γ-TuSC$^{Alp6\text{-}MBP}$:Mzt1 may have a small amount of Alp6 that is not in complex with other γ-TuSC proteins. In E, the relative stoichiometry of MGM proteins is essentially constant across the gradient. A small excess of Alp6 in the first few fractions is likely due to the reason mentioned above. In this analysis, Mto2 was not quantified because its multiple bands (due to multiple phosphorylation states; [S1]) are less distinct and are close to Gtb1**.** **(F)** Negative-stain electron microscopy of MGM purified from coexpression of Mto1/2[bonsai] and γ-TuSC$^{Alp6\text{-}MBP}$:Mzt1. Complete uncropped image fields are shown. Image shown in Figure 2D is taken from center of field on left. Field on right has higher contrast but worse preservation of morphology.

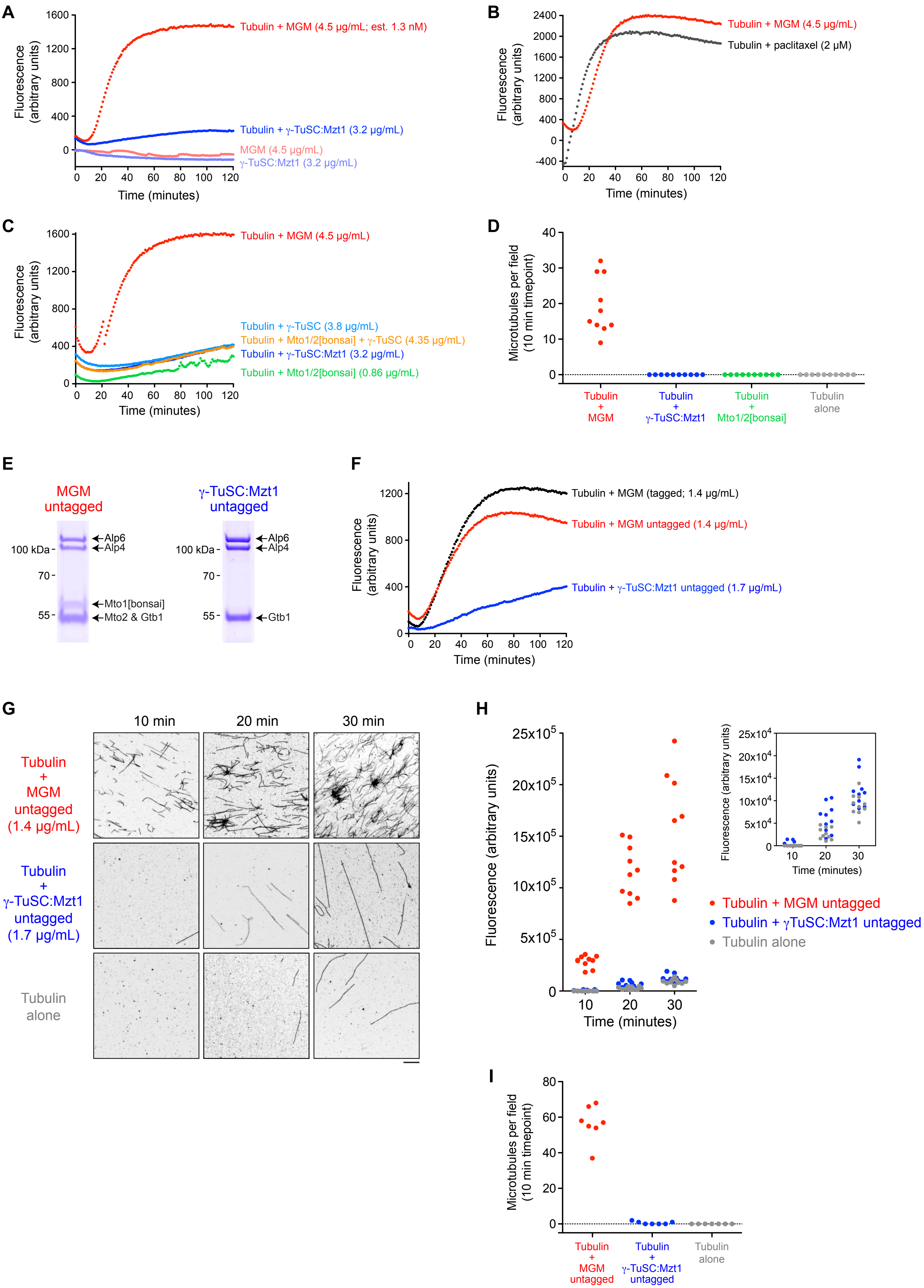

A
Tubulin + MGM (4.5 µg/mL; est. 1.3 nM)
Tubulin + γ-TuSC:Mzt1 (3.2 µg/mL)
MGM (4.5 µg/mL)
γ-TuSC:Mzt1 (3.2 µg/mL)
Fluorescence (arbitrary units)
Time (minutes)
B
Tubulin + MGM (4.5 µg/mL)
Tubulin + paclitaxel (2 µM)
C
Tubulin + MGM (4.5 µg/mL)
Tubulin + γ-TuSC (3.8 µg/mL)
Tubulin + Mto1/2[bonsai] + γ-TuSC (4.35 µg/mL)
Tubulin + γ-TuSC:Mzt1 (3.2 µg/mL)
Tubulin + Mto1/2[bonsai] (0.86 µg/mL)
D
Microtubules per field (10 min timepoint)
Tubulin + MGM
Tubulin + γ-TuSC:Mzt1
Tubulin + Mto1/2[bonsai]
Tubulin alone
E
MGM untagged
γ-TuSC:Mzt1 untagged
Alp6
Alp4
Mto1[bonsai]
Mto2 & Gtb1
Gtb1
100 kDa
70
55
F
Tubulin + MGM (tagged; 1.4 µg/mL)
Tubulin + MGM untagged (1.4 µg/mL)
Tubulin + γ-TuSC:Mzt1 untagged (1.7 µg/mL)
G
10 min
20 min
30 min
Tubulin + MGM untagged (1.4 µg/mL)
Tubulin + γ-TuSC:Mzt1 untagged (1.7 µg/mL)
Tubulin alone
H
Tubulin + MGM untagged
Tubulin + γTuSC:Mzt1 untagged
Tubulin alone
I
Tubulin + MGM untagged
Tubulin + γ-TuSC:Mzt1 untagged

**Figure S3. Additional controls for assays of microtubule nucleation by MGM holocomplex. Related to Figure 3.**
**(A)** DAPI fluorescence assay showing that in the absence of tubulin, neither MGM nor $\gamma$-TuSC$^{Alp6\text{-}MBP}$:Mzt1 causes an increase in fluorescence. This indicates that fluorescence increase is due to microtubule (MT) polymerization. Data for tubulin plus MGM and tubulin plus $\gamma$-TuSC$^{Alp6\text{-}MBP}$:Mzt1 are reproduced from Figure 3A. **(B)** DAPI fluorescence assay for MT polymerization, comparing activity of MGM holocomplex with 2 µM paclitaxel (positive control for MT assembly). **(C)** DAPI fluorescence assay showing that subcomplexes of MGM are insufficient for MT nucleation. Different "µg/mL" concentrations were used in order to have comparable molarities for the different protein complexes. Discontinuity in red trace was due to an air bubble. **(D)** Quantification of MT number (MTs per microscope field) for experiments shown in Figure 3B. Only the 10-minute timepoint is shown, because MT bundling or clumping at later timepoints precluded accurate counts in most fields. **(E)** SDS-PAGE of MGM and $\gamma$-TuSC:Mzt1 after removal of MBP-6xHis tag from Alp6-MBP and 6xHis-tags from Mto1[bonsai] and Mzt1 proteins. The resulting untagged complexes were used for experiments in (F-H). **(F)** DAPI fluorescence assay showing that untagged MGM and tagged MGM have comparable effects on MT nucleation. **(G)** Rhodamine-tubulin fluorescence microscopy assay for MT nucleation, using the indicated untagged complexes at the same concentrations as in F. **(H)** Quantification of MT polymerization for experiments in G. Each data point represents total MT fluorescence within a randomly-chosen field. Inset shows expanded scale for tubulin plus untagged $\gamma$-TuSC:Mzt1 and for tubulin alone. **(I)** Quantification of MT number (MTs per microscope field) for experiments in G. Only the 10-minute timepoint is shown. Note that experiments in F-H used a different batch of tubulin compared to those shown in A-D and in Figure 3; therefore, results from the two sets of experiments should not be compared directly. Scale bar, 10 µm.

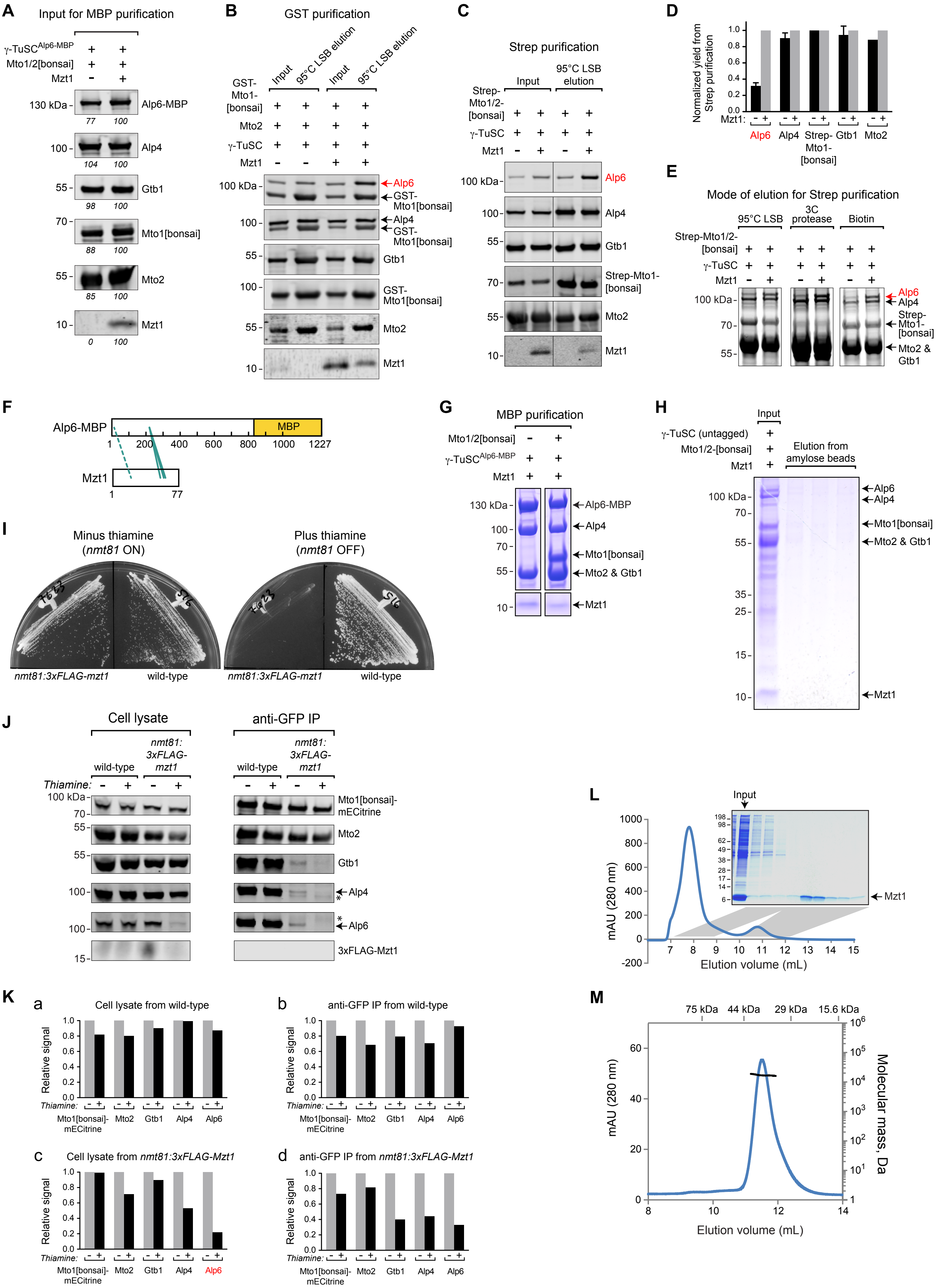
A
Input for MBP purification
γ-TuSC^Alp6-MBP
Mto1/2[bonsai]
Mzt1
130 kDa
Alp6-MBP
77 100
Alp4
104 100
Gtb1
98 100
Mto1[bonsai]
88 100
Mto2
85 100
Mzt1
0 100
B
GST purification
Input
95°C LSB elution
GST-Mto1-[bonsai]
Mto2
γ-TuSC
Mzt1
Alp6
GST-Mto1[bonsai]
Alp4
Gtb1
Mto2
Mzt1
C
Strep purification
Input
95°C LSB elution
Strep-Mto1/2-[bonsai]
γ-TuSC
Mzt1
Alp6
Alp4
Gtb1
Strep-Mto1-[bonsai]
Mto2
Mzt1
D
Normalized yield from Strep purification
Mzt1:
Alp6
Alp4
Strep-Mto1-[bonsai]
Gtb1
Mto2
E
Mode of elution for Strep purification
95°C LSB
3C protease
Biotin
Strep-Mto1/2-[bonsai]
γ-TuSC
Mzt1
Alp6
Alp4
Strep-Mto1-[bonsai]
Mto2 & Gtb1
F
Alp6-MBP
MBP
1 200 400 600 800 1000 1227
Mzt1
1 77
G
MBP purification
Mto1/2[bonsai]
γ-TuSC^Alp6-MBP
Mzt1
Alp6-MBP
Alp4
Mto1[bonsai]
Mto2 & Gtb1
Mzt1
H
Input
γ-TuSC (untagged)
Mto1/2-[bonsai]
Mzt1
Elution from amylose beads
Alp6
Alp4
Mto1[bonsai]
Mto2 & Gtb1
Mzt1
I
Minus thiamine (nmt81 ON)
Plus thiamine (nmt81 OFF)
nmt81:3xFLAG-mzt1
wild-type
nmt81:3xFLAG-mzt1
wild-type
J
Cell lysate
anti-GFP IP
wild-type
nmt81: 3xFLAG-mzt1
Thiamine:
Mto1[bonsai]-mECitrine
Mto2
Gtb1
Alp4
Alp6
3xFLAG-Mzt1
K
a
Cell lysate from wild-type
b
anti-GFP IP from wild-type
c
Cell lysate from nmt81:3xFLAG-Mzt1
d
anti-GFP IP from nmt81:3xFLAG-Mzt1
Relative signal
Thiamine:
Mto1[bonsai]-mECitrine
Mto2
Gtb1
Alp4
Alp6
L
Input
mAU (280 nm)
Elution volume (mL)
Mzt1
M
75 kDa 44 kDa 29 kDa 15.6 kDa
mAU (280 nm)
Molecular mass, Da
Elution volume (mL)

**Figure S4. Additional data supporting a role for Mzt1 in stabilizing Alp6 within the MGM holocomplex. Related to Figure 4.**

**(A)** Western blots of insect cell lysates used as inputs for MBP purifications shown in Figure 4A. Numbers in italics show quantification of western blots, normalized to the signal in the presence of Mzt1 (set to 100). Mto1[bonsai] and Mto2 levels are similar in presence and absence of Mzt1. **(B)** Western blots used for quantification in Figure 4D. Because anti-Alp6 and anti-Alp4 antisera were raised against GST-fusion proteins, they also recognize GST-Mto1[bonsai]. (Affinity-purified antibodies were used only when needed to avoid excessive background bands, because affinity purification led to low yields and decreased titer.) **(C)** Western blots of insect cell lysates and elutions (95°C in Laemmli sample buffer; LSB) from Strep-Tactin-based purification of coexpressed Mto1/2[bonsai]$^{\text{Strep-Mto1[bonsai]}}$ and γ-TuSC in the absence vs. presence of Mzt1. (**D)** Quantification of western blots of the type shown in C. Error bars indicate SEM, based on multiple replicate experiments (see STAR Methods). Alp6 is not copurified as efficiently as other proteins. **(E)** SDS-PAGE of Strep-Tactin-based purification of coexpressed Mto1/2[bonsai]$^{\text{Strep-Mto1[bonsai]}}$ and γ-TuSC in the absence vs. presence of Mzt1 (SYPRO Ruby stain). In parallel experiments, proteins were eluted from Strep-Tactin beads either by heating at 95°C in LSB, by cleavage of the Strep-tag from Mto1[bonsai] using rhinovirus 3C protease, or by biotin elution. In all cases, absence of Mzt1 coexpression leads to decreased copurification of Alp6 (e.g. compared to Alp4). Additional ~50 kDa band in 3C protease elution is 3C protease itself (fused to GST). **(F)** Crosslinks between Mzt1 and Alp6-MBP identified by mass spectrometry. Solid lines indicate EDC crosslinks from Alp6 Ser219 to Mzt1 Glu62, and from Alp6 Lys220 to Mzt1 Glu56, Asp60 and Glu62. A lower-scoring EDC crosslink from Alp6 Lys307 to Mzt1 Asp60 is not shown. Dashed line indicates Sulfo-SDA crosslink from Alp6 Leu13 to Mzt1 Thr21. **(G)** SDS-PAGE of amylose purification after coexpression of the indicated proteins. Copurification of Mzt1 with γ-TuSC$^{\text{Alp6-MBP}}$ is not increased by the presence of Mto1/2[bonsai]. **(H)** SDS-PAGE showing that when Mto1/2[bonsai] and γ-TuSC:Mzt1 are coexpressed without MBP tag, no MGM proteins are purified on amylose (three right-most lanes represent successive column elutions). This is a negative control for experiment in Figure 4H. **(I)** Growth of wild-type and *nmt81:3xFLAG-mzt1* fission yeast (strains KS516 and KS7623, respectively) after two days incubation (32°C) in absence or presence of thiamine. In absence of thiamine (*nmt81* promoter derepressed), the two strains grow identically. In presence of thiamine (*nmt81* promoter repressed), *nmt81:3xFLAG*-mzt1 show negligible growth. **(J)** Western blots of clarified cell lysates and anti-GFP immunoprecipitates (IPs) from *mto1[bonsai]-mECitrine* fission yeast cells expressing either wild-type Mzt1 or *nmt81:*3xFLAG-Mzt1, in absence or presence of thiamine. Lanes for cell lysates contain 1% of the "cell-equivalents" used in lanes for anti-GFP IPs. For a given protein, the same contrast setting was used for lysates and anti-GFP IPs. Asterisks indicate non-specific bands. **(K)** Quantification of Western blots from (J). Within each graph, signals for a given protein were normalized to the signal in absence of thiamine. Three points should be noted from results in I-K. First, repression of *nmt81:3xFLAG-Mzt1* expression leads to decreased levels of Alp6 (and to some extent, Alp4) in cell lysates (graph a in K). This suggests that Mzt1 contributes to Alp6 stabilization, consistent with *in vitro* experiments. Second, repression of *nmt81:3xFLAG-Mzt1* accordingly leads to decreased coIP of γ-TuSC proteins with Mto1[bonsai]-mECitrine (graph b in K). Third, even under derepressing conditions, *nmt81:3xFLAG-Mzt1* cells show significantly decreased coIP of γ-TuSC proteins with Mto1[bonsai]-mECitrine compared to wild-type cells grown under the same conditions (IP lanes in J). This suggests that *nmt81:3xFLAG-Mzt1* is not completely functional. While this last point underscores the importance of Mzt1 for the γ-TuSC-Mto1/2[bonsai] interaction, it was also somewhat unexpected, because *nmt81:3xFLAG-Mzt1* cells grow well under depressing conditions; by contrast, *mzt1Δ* cells are inviable [S2, S3]. One explanation for this result is that *nmt81:3xFLAG-Mzt1* function, although impaired, is above the threshold required for function of γ-TuSC with Pcp1, a paralog of Mto1 that is required for intranuclear mitotic spindle formation and is essential for viability [S4, S5]. By contrast, Mto1 is required for all microtubule nucleation in the cytoplasm (and thus for normal cell morphology) but is not essential for viability [S6, S7]. **(L)** Superdex 75 size-exclusion chromatography of Mzt1 alone (without γ-TuSC). **(M)** SEC-MALS analysis of purified Mzt1. Molecular mass (19.6 kDa) indicates that Mzt1 is a dimer in solution.

| Oligonucleotide | Source | Identifier |
|---|---|---|
| *PCR forward primer to generate insect cell expressed Mzt-6xHis:* 5'-GGGCTCGAGGGATCCATGTCCGAGTCCAC-3' | Eurofins | OKS2838 |
| *PCR reverse primer to generate insect cell expressed Mzt-6xHis:* 5'-GCTAGCCTGCAGTTAGTGATGATGATGATGATGACCCTGAAAGTAAAGATTTTCGCTGGGCTC-3' | Eurofins | OKS2839 |
| *PCR forward primer to generate insect cell expressed 6xHis-Mto1[bonsai]:* 5'-GGGGCCATGGGACATCATCATCATCATCACGAAAATCTTTACTTTCAGGGTACTACTGATGATGAACAGCTT-3' | Eurofins | OKS2182 |
| *PCR reverse primer to generate insect cell expressed 6xHis-Mto1[bonsai]:* 5'-GGTCATGCATTTATTACTGGAGTTCTTGGGTAAT-3' | Eurofins | OKS2184 |
| *PCR forward primer to generate insect cell expressed Mto2:* 5'-GGGGTGATCAATGTCTGAACATAATTACCAG-3' | Eurofins | OKS2185 |
| *PCR reverse primer to generate insect cell expressed Mto2 and 6xHis-Mto2:* 5'-GGTCGTCGACTTACTAGGGGGAAGGAGTGTCTTG-3' | Eurofins | OKS2186 |
| *PCR forward primer to generate insect cell expressed 6xHis-Mto2:* 5'-GGGGTGATCAATGCATCATCATCATCATCACGAAAATCTTTACTTTCAGGGTTCTGAACATAATTACCAGTCT-3' | Eurofins | OKS2320 |
| *PCR forward primer to generate bacterial GST-Mzt-6xHis with Gateway cloning:* 5'-GGGGACAAGTTTGTACAAAAAAGCAGGCTGGATCCGGATGGAGAACCTCTACTTCCAAGGTATGAGCGAAAGCACCAAAGAAACCATTGA-3' | Eurofins | OKS2874 |
| *PCR reverse primer to generate bacterial GST-Mzt-6xHis with Gateway cloning:* 5'-GGGGACCACTTTGTACAAGAAAGCTGGGTCCTGCAGGGCGCCGGGCCCCTGGAACAGAACTTCCAGGCTCGGTTCGGTATCAACGGTCTG-3' | Eurofins | OKS2875 |
| *PCR forward primer to generate bacterial Mzt-6xHis with Gateway cloning:* 5'-GGGGACAAGTTTGTACAAAAAAGCAGGCTGGATCCGGATGAGCGAAAGCACCAAAGAAACCATTGAAGT-3' | Eurofins | OKS2876 |
| *PCR reverse primer to generate bacterial Mzt-6xHis with Gateway cloning:* 5'-GGGGACCACTTTGTACAAGAAAGCTGGGTCCTGCAGGGCGCCGGGCCCCTGGAACAGAACTTCCAGGCTCGGTTCGGTATCAACGGTCTG-3' | Eurofins | OKS2875 |
| *PCR forward primer 1 for N-terminal Strep-tag of Mto1[bonsai]:* 5'-gggCTCGAGatgGGAAGCGCTTGGAGCCACCCGCAGTTCGAAAAAGGTGGAGGTTCTGGCGGTGGATCGGGAGGTTCAGCGTGGAGCCACCC-3' | Eurofins | OKS2883 |
| *PCR forward primer 2 for N-terminal Strep-tag of Mto1[bonsai]:* 5'-GGGAGGTTCAGCGTGGAGCCACCCGCAGTTCGAGAAACTGGAAGTTCTGTTCCAGGGGCCCACTACTGATGATGAACAGCTTTCTCCCGTA-3' | Eurofins | OKS2884 |
| *PCR reverse primer for N-terminal Strep-tag of Mto1[bonsai]:* 5'-ACCGCATGCTATGCATTTATTACTGGAGT-3' | Eurofins | OKS2978 |
| *PCR forward primer for tagging Mzt1 with nmt81:3xFLAG:* 5'-CCTATTCCAGTAAACTTAACATTACCAACTTGTTACGAAGTATATATAATTAGACTAGTTTATTATAAAGTATCGAGTTTTGAATTCGAGCTCGTTTAAAC-3' | Eurofins | OKS2671 |
| *PCR reverse primer for tagging Mzt1 with nmt81:3xFLAG:* 5'-CGTTGTTTTATCAAGTTCAGTTCCAAGCAATGTTCCTATTTCGTATAAAACTTCTATTGTCTCTTTGGTAGATTCAGACATTCCGCCTCCTTTATCATCATCGTCCTTATAG-3' | Eurofins | OKS2672 |
| *PCR forward primer for tagging Mto1[bonsai] with mECitrine:* 5'-TGATGAGAATGGAGCAACAATGGCGGGAAGATGTTGACCAACTCCAGGAATATGTCGAAGAGATTACCCAAGAACTCCAGCGGATCCCCGGGTTAATTAA-3' | Eurofins | OKS2105 |
| *PCR reverse primer for tagging Mto1[bonsai] with mECitrine:* 5'-TATAAATCTTGGAATTAGCTTATTTTGTCAATTAAATTCTCCAACTAAGTAGATATGTACTGGTGATTAAGAAAAGCTATGAATTCGAGCTCGTTTAAAC-3' | Eurofins | OKS2116 |

**Table S1. Oligonucleotides used in this work. Related to STAR Methods and Key Resources Table.**

**Supplemental References**


S1. Borek, W.E., Groocock, L.M., Samejima, I., Zou, J., de Lima Alves, F., Rappsilber, J., and Sawin, K.E. (2015). Mto2 multisite phosphorylation inactivates non-spindle microtubule nucleation complexes during mitosis. Nat Commun *6*, 7929.

S2. Dhani, D.K., Goult, B.T., George, G.M., Rogerson, D.T., Bitton, D.A., Miller, C.J., Schwabe, J.W., and Tanaka, K. (2013). Mzt1/Tam4, a fission yeast MOZART1 homologue, is an essential component of the gamma-tubulin complex and directly interacts with GCP3(Alp6). Mol Biol Cell *24*, 3337-3349.

S3. Masuda, H., Mori, R., Yukawa, M., and Toda, T. (2013). Fission yeast MOZART1/Mzt1 is an essential gamma-tubulin complex component required for complex recruitment to the microtubule organizing center, but not its assembly. Mol Biol Cell *24*, 2894-2906.

S4. Flory, M.R., Morphew, M., Joseph, J.D., Means, A.R., and Davis, T.N. (2002). Pcp1p, an Spc110p-related calmodulin target at the centrosome of the fission yeast Schizosaccharomyces pombe. Cell Growth Differ *13*, 47-58.

S5. Fong, C.S., Sato, M., and Toda, T. (2010). Fission yeast Pcp1 links polo kinase-mediated mitotic entry to gamma-tubulin-dependent spindle formation. EMBO J *29*, 120-130.

S6. Sawin, K.E., Lourenco, P.C., and Snaith, H.A. (2004). Microtubule nucleation at non-spindle pole body microtubule-organizing centers requires fission yeast centrosomin-related protein mod20p. Curr Biol *14*, 763-775.

S7. Venkatram, S., Tasto, J.J., Feoktistova, A., Jennings, J.L., Link, A.J., and Gould, K.L. (2004). Identification and characterization of two novel proteins affecting fission yeast gamma-tubulin complex function. Mol Biol Cell *15*, 2287-2301.
