# Supplementary material for: Reconstitution of Microtubule Nucleation In Vitro Reveals Novel Roles for Mzt1
Source: Curr Biol. 2019 Jul 8;29(13):2199–2207.e10. doi: 10.1016/j.cub.2019.05.058 (PMC6616311; doi:10.1016/j.cub.2019.05.058)
Supplement: Document S2. Article plus Supplemental Information [file mmc2.pdf]

Report

Current Biology

Reconstitution of Microtubule Nucleation *In Vitro* Reveals Novel Roles for Mzt1

Graphical Abstract

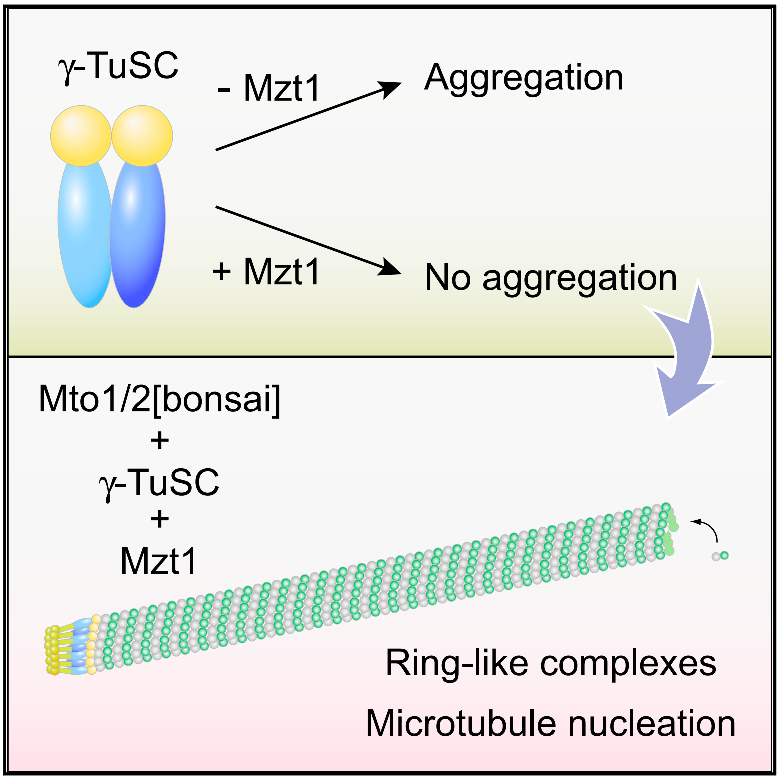

Highlights

- Mto1/2[bonsai] complex, γ-tubulin small complex, and Mzt1 form "MGM" holocomplex
- MGM holocomplex has a ring-like structure
- MGM holocomplex nucleates microtubules *in vitro*
- Mzt1 stabilizes Alp6 (GCP3 homolog) within MGM holocomplex.

Authors

Su Ling Leong, Eric M. Lynch, Juan Zou, ..., Maarten W. Tuijtel, Juri Rappsilber, Kenneth E. Sawin

Correspondence

ken.sawin@ed.ac.uk

In Brief

Leong et al. reconstitute microtubule nucleation *in vitro* with a minimal set of purified fission yeast proteins—Mto1/2 [bonsai] complex, heterotetrameric γ-TuSC, and Mzt1—that assemble into a ring-like "MGM" holocomplex. The small conserved protein Mzt1 is implicated in stabilization of Alp6, fission yeast ortholog of mammalian γ-TuSC protein GCP3.

Leong et al., 2019, Current Biology *29*, 2199–2207
July 8, 2019 © 2019 The Authors. Published by Elsevier Ltd.
https://doi.org/10.1016/j.cub.2019.05.058

CellPress

Current Biology
Report

Reconstitution of Microtubule Nucleation *In Vitro* Reveals Novel Roles for Mzt1

Su Ling Leong,[1] Eric M. Lynch,[1,3] Juan Zou,[1] Ye Dee Tay,[1] Weronika E. Borek,[1] Maarten W. Tuijtel,[1] Juri Rappsilber,[1,2] and Kenneth E. Sawin[1,4,*]
[1]Wellcome Centre for Cell Biology, School of Biological Sciences, University of Edinburgh, Michael Swann Building, Max Born Crescent, Edinburgh EH9 3BF, UK
[2]Chair of Bioanalytics, Institute of Biotechnology, Technische Universität Berlin, Berlin 13355, Germany
[3]Present address: Department of Biochemistry, University of Washington, Seattle, Washington 98195, USA
[4]Lead Contact
*Correspondence: ken.sawin@ed.ac.uk
https://doi.org/10.1016/j.cub.2019.05.058

SUMMARY

Microtubule (MT) nucleation depends on the γ-tubulin complex (γ-TuC), in which multiple copies of the heterotetrameric γ-tubulin small complex (γ-TuSC) associate to form a ring-like structure (in metazoans, γ-tubulin ring complex; γ-TuRC) [1–7]. Additional conserved regulators of the γ-TuC include the small protein Mzt1 (MOZART1 in human; GIP1/1B and GIP2/1A in plants) [8–13] and proteins containing a Centrosomin Motif 1 (CM1) domain [10, 14–19]. Many insights into γ-TuC regulators have come from *in vivo* analysis in fission yeast *Schizosaccharomyces pombe*. The *S. pombe* CM1 protein Mto1 recruits the γ-TuC to microtubule-organizing centers (MTOCs) [14, 20–22], and analysis of Mto1[bonsai], a truncated version of Mto1 that cannot localize to MTOCs, has shown that Mto1 also has a role in γ-TuC activation [23]. *S. pombe* Mzt1 interacts with γ-TuSC and is essential for γ-TuC function and localization to MTOCs [11, 12]. However, the mechanisms by which Mzt1 functions remain unclear. Here we describe reconstitution of MT nucleation using purified recombinant Mto1[bonsai], the Mto1 partner protein Mto2, γ-TuSC, and Mzt1. Multiple copies of the six proteins involved coassemble to form a 34-40S ring-like "MGM" holocomplex that is a potent MT nucleator *in vitro*. Using purified MGM and subcomplexes, we investigate the role of Mzt1 in MT nucleation. Our results suggest that Mzt1 is critical to stabilize Alp6, the *S. pombe* homolog of human γ-TuSC protein GCP3, in an "interaction-competent" form within the γ-TuSC. This is essential for MGM to become a functional nucleator.

RESULTS AND DISCUSSION

Mzt1 Prevents Large-Scale Aggregation of the Fission Yeast γ-TuSC *In Vitro*

S. pombe γ-TuSC contains the proteins γ-tubulin, Alp4 (homolog of human GCP2; see Figure S1A for protein nomenclature), and Alp6 (homolog of human GCP3) in a 2:1:1 ratio [24]. We purified γ-TuSC using proteins expressed in insect cells and an MBP affinity tag on Alp6 (Figure 1A). After density-gradient centrifugation, γ-TuSC$^{Alp6\text{-}MBP}$ was present mainly in the pellet fraction, with a sedimentation coefficient of at least ~150S, indicating large, non-physiological aggregates (Figures 1C and S1C). Consistent with this, in size-exclusion chromatography (SEC) on Superose 6, some γ-TuSC$^{Alp6\text{-}MBP}$ was not recovered from the column, and the remainder eluted mainly in the void volume (Figure 1B).

Next, we coexpressed γ-TuSC$^{Alp6\text{-}MBP}$ proteins with Mzt1. Mzt1 is conserved in animals, plants, and many fungi, although not in the group of Saccharomycotina that includes budding yeast *S. cerevisiae* [10]. In *S. pombe*, Mzt1 is an essential component of the γ-TuC and interacts with Alp6 [11, 12]. Mzt1 copurified with γ-TuSC$^{Alp6\text{-}MBP}$, as a γ-TuSC$^{Alp6\text{-}MBP}$:Mzt1 complex (Figures 1D and S1B). Interestingly, upon density-gradient centrifugation, relatively little γ-TuSC$^{Alp6\text{-}MBP}$:Mzt1 complex was pelleted; instead, the complex showed a sedimentation peak at ~24S, with a broad overall sedimentation profile relative to S-value standards (Figures 1F, S1D, and S2A). Because γ-TuSCs from *S. cerevisiae* and *Drosophila* typically sediment at ~10–12S [25–27], this suggests that under our experimental conditions, γ-TuSC$^{Alp6\text{-}MBP}$:Mzt1 may coexist in several lower-order oligomerization states.

In SEC on Superose 6, although some γ-TuSC$^{Alp6\text{-}MBP}$:Mzt1 was not recovered from the column, most of the γ-TuSC$^{Alp6\text{-}MBP}$:Mzt1 that did elute from the column eluted at a position expected for γ-TuSCs, and a much smaller proportion eluted in the void volume (Figure 1E).

We conclude that Mzt1 binds stably to γ-TuSC and prevents its aggregation *in vitro*. The presence of TuSC:Mzt1 oligomers on density gradients raises the possibility that Mzt1 may also contribute to γ-TuSC-γ-TuSC interactions. However, because γ-TuSC aggregates in the absence of Mzt1, it is equally possible that the effect of Mzt1 on γ-TuSC oligomerization is indirect.

Mto1/2[bonsai] Complex Interacts with γ-TuSC:Mzt1 to Form an "MGM" Holocomplex

Mto1[bonsai] lacks N- and C-terminal residues (1–130 and 550–1,115, respectively) that target Mto1 to MTOCs [21, 22]. However, Mto1[bonsai] still interacts with the Mto1 partner protein Mto2 to generate an Mto1/2[bonsai] complex [23]. *In vivo*, Mto1/2[bonsai]

This is an open access article under the CC BY license (http://creativecommons.org/licenses/by/4.0/).

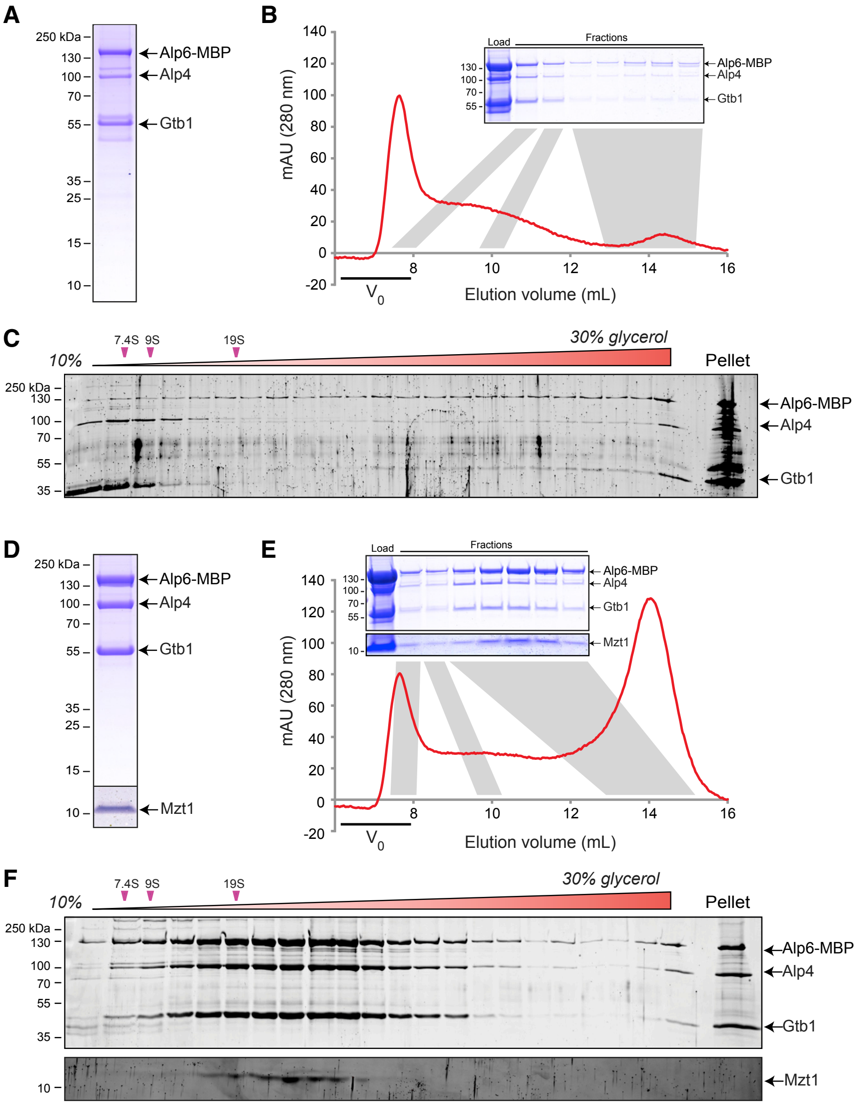

Figure 1. Mzt1 Prevents Large-Scale Aggregation of the γ-TuSC *In Vitro*

(A) SDS-PAGE of γ-TuSC$^{Alp6-MBP}$ (without Mzt1), purified on amylose via Alp6-MBP.

(B) Superose 6 size-exclusion chromatography (SEC) of γ-TuSC$^{Alp6-MBP}$, with corresponding SDS-PAGE of indicated fractions. The majority of eluting γ-TuSC$^{Alp6-MBP}$ elutes in the void volume.

(C) SDS-PAGE of 80-min density-gradient centrifugation of γ-TuSC$^{Alp6-MBP}$ (SYPRO Ruby stain). Most γ-TuSC is in the pellet. Results from a 45-min centrifugation are shown in Figure S1C.

(D) SDS-PAGE of γ-TuSC$^{Alp6-MBP}$ with Mzt1 (γ-TuSC$^{Alp6-MBP}$:Mzt1), purified as above. Mzt1 is shown from a higher-contrast image, shown in Figure S1B.

(E) Superose 6 SEC of (γ-TuSC$^{Alp6-MBP}$:Mzt1), with corresponding SDS-PAGE of indicated fractions. Alp6-MBP, Alp4, γ-tubulin (Gtb1), and Mzt1 coelute, much later than the void volume.

(F) SDS-PAGE of 80-min density-gradient centrifugation of γ-TuSC$^{Alp6-MBP}$:Mzt1 (SYPRO Ruby stain). γ-TuSC$^{Alp6-MBP}$:Mzt1 sediments with a broad profile centered at ~24S. Mzt1 was visualized on a separate gel with higher acrylamide concentration. Results from a 45-min centrifugation are shown in Figure S1D.

See also Figure S1.

forms puncta that contain γ-TuSCs and nucleate single MTs [23]. To determine if Mto1/2[bonsai] interacts directly with γ-TuSC *in vitro*, we purified the Mto1/2[bonsai] complex from insect-cell expression and combined it with γ-TuSC$^{Alp6-MBP}$:Mzt1. Upon density-gradient centrifugation, Mto1/2[bonsai] alone sedimented with a peak at 10S and a long "tail" at higher S-values, suggesting multiple oligomerization states (Figures 2A and S2A). Combining Mto1/2[bonsai] with γ-TuSC$^{Alp6-MBP}$:Mzt1 led to the formation of a new holocomplex, in which all components of both complexes cosedimented, with a new peak at 34–40S (Figures 2B, S2A, and S2D). Like the individual complexes, the holocomplex had a broad sedimentation profile relative to S-value standards (Figure S2A); we speculate that this could be due to conformational heterogeneity and/or small variations in protein copy number within the holocomplex. We will refer to the Mto1/2[bonsai]:γ-TuSC:Mzt1 holocomplex as the "MGM" (Mto/Gamma/Mozart) holocomplex. We also generated the MGM holocomplex via coexpression of Mto1/2[bonsai] with γ-TuSC$^{Alp6-MBP}$:Mzt1 (Figure S2B). MGM purified from coexpression, which had essentially the same composition and sedimentation properties as MGM obtained by combining separate complexes (Figures S2C and S2E), was used in subsequent experiments.

We investigated protein-protein contacts within the MGM holocomplex by chemical crosslinking mass spectrometry (CLMS), using the zero-length crosslinker 1-ethyl-3-(3-dimethylaminopropyl)carbodiimide (EDC) (Figure 2C). Although this analysis was not exhaustive, we observed crosslinks between Alp4 and Alp6 along the length of these two proteins, consistent with their general parallel lateral alignment in current models for γ-TuC organization [1]. In addition, we observed specific crosslinks from both Alp4 and Alp6 to the Mto1 [bonsai] CM1 domain and/or its immediate flanking regions. Interestingly, crosslinks from Alp4 and Alp6 N-terminal regions tended to be to the C-terminal portion of the CM1 domain, while crosslinks from Alp4 and Alp6 C-terminal regions tended to be to the N-terminal portion of the CM1 domain. This raises the possibility that the CM1 domain, which is adjacent to coiled-coil regions, may be oriented antiparallel to Alp4 and Alp6.

Imaging of the MGM holocomplex by negative-stain electron microscopy revealed a range of ring- and crescent-like structures with a diameter of ~25 nm, reminiscent of isolated metazoan γ-TuRC (Figures 2D and S2F [28, 29]). These features suggest that MGM may have an overall structure similar to γ-TuRC; further work may be required to optimize morphological preservation.

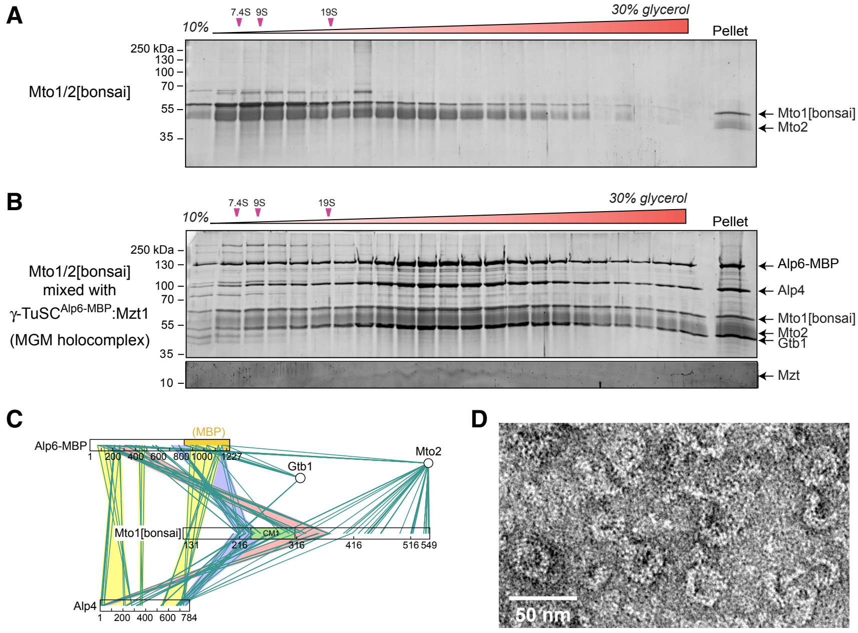

Figure 2. γ-TuSC:Mzt1 Interacts with Mto1/2[bonsai] Complex to Form An "MGM" (Mto/Gamma/Mzt) Holocomplex

(A) SDS-PAGE of 80-min density-gradient centrifugation of Mto1/2[bonsai].

(B) SDS-PAGE of 80-min density-gradient centrifugation of Mto1/2[bonsai] mixed (after purification) with γ-TuSC$^{Alp6\text{-}MBP}$:Mzt1. Mixing alters the sedimentation profiles of both complexes, and all constituent proteins cosediment. Mzt1 was visualized on a separate gel with higher acrylamide concentration. Gels are stained with SYPRO Ruby. Representative sedimentation profiles from (A) and (B) are shown in Figure S2A.

(C) Interactions between different proteins within MGM holocomplex, identified by zero-length chemical crosslinking and mass spectrometry (see STAR Methods). For simplicity, only inter-protein crosslinks are shown. In this experiment, crosslinks to Mzt1 (a very small protein with few useful proteolytic fragments) were not identified.

(D) Negative-stain electron microscopy of MGM. The image is a portion of a field shown in Figure S2F. In (C) and (D), MGM was purified from coexpression of Mto1/2[bonsai] and γ-TuSC$^{Alp6\text{-}MBP}$:Mzt1 proteins.

See also Figures 1 and S2.

The MGM Holocomplex Is a Potent Microtubule Nucleator *In Vitro*

To determine if the MGM holocomplex is sufficient for MT nucleation *in vitro*, we tested its ability to promote polymerization of porcine brain tubulin at tubulin concentrations below the critical concentration for spontaneous assembly, without any further additives to promote MT assembly. We first assayed polymerization by measuring fluorescence of 4',6-diamidino-2-phenylindole (DAPI) bound to MT polymer [30]. At a concentration of 4.5 μg/mL (estimated to be ~1.3 nM; see STAR Methods), MGM holocomplex promoted tubulin polymerization to a level comparable to that obtained by incubating tubulin with paclitaxel, although the rate of paclitaxel-induced polymerization was somewhat faster (Figures 3A and S3B). At a 10-fold lower concentration (i.e., estimated ~130 pM), MGM promoted polymerization more slowly and to a lesser extent (Figure 3A); this indicates that MGM-induced polymerization is dose dependent. Assays of subcomplexes lacking specific components of the MGM holocomplex showed that the full complement of MGM proteins is required for activity (Figures 3A and S3C).

To confirm that increased DAPI fluorescence represented bona fide MTs, we assayed MGM holocomplex activity using rhodamine-tubulin and fluorescence microscopy (Figures 3B, 3C, and S3D). MGM promoted the formation of morphologically normal MTs, while subcomplexes did not promote polymerization significantly beyond that seen with tubulin alone.

Because MGM used in MT nucleation experiments contained MBP-tagged Alp6, and CLMS experiments suggested that the MBP moiety is spatially close to γ-tubulin (Figure 2C), we confirmed that MBP was not artifactually promoting MGM function. After TEV cleavage and removal of MBP, MGM remained active in polymerization assays (Figures S3E–S3I). We conclude that the MGM holocomplex is an MT nucleator *in vitro*.

Relative to recently reconstituted MT nucleation complexes using *S. cerevisiae* and *C. albicans* proteins [10, 31], MGM appears to be a potent nucleator. The *S. cerevisiae* complex (i.e., γ-TuSC plus an N-terminal fragment of the CM1 protein Spc110) shows a strong preference for *S. cerevisiae* tubulin and is only weakly active on mammalian tubulin, and nucleation assays for this complex included glycerol to aid MT assembly [31]. Nucleation assays for the *C. albicans* complex (i.e., *Ca*γ-TuSC, *Ca*Mzt1, and an N-terminal fragment of either of the CM1 proteins *Ca*Spc110 or *Ca*Spc72) used porcine brain tubulin but also included glycerol, as well as low concentrations of paclitaxel, supposedly to counteract species-specific differences between *Ca*γ-TuSC and mammalian tubulin [10]. In addition, the *C. albicans* complex was used at relatively high concentrations (~80 nM, based on the same estimation procedure that we applied to MGM). Although different assay conditions make detailed comparisons difficult, one possible reason that MGM may be a particularly good nucleator is that in addition to Mto1[bonsai], it also contains Mto2. Mto2 appears to promote Mto1 multimerization *in vivo* [23], and Mto2 could also potentially serve to orient Mto1 CM1 domains relative to γ-TuSCs.

MT nucleation *in vitro* by a ring-like 34-40S MGM holocomplex is consistent with our previous characterization of single-MT nucleation *in vivo* by puncta that contain Mto1/2[bonsai] and γ-TuSC in copy numbers similar to that in γ-TuRC [23]. Reconstitution of nucleation using only Mto1/2[bonsai], γ-TuSC, and Mzt1 also extends previous findings that *S. pombe* homologs of human GCP4, GCP5 and GCP6 (Gfh1, Mod21, and Alp16, respectively) are not essential for nucleation, although they do contribute to overall MT polymerization *in vivo* [32, 33].

Mzt1 Stabilizes Alp6 in an "Interaction-Competent" State within Higher-Order Complexes

We next investigated the role of Mzt1 within the MGM holocomplex. By comparing different tag-based purifications of

coexpressed MGM proteins in the absence versus presence of Mzt1, we found that Mzt1 is required for Alp6 and Mto1/2[bonsai] to be mutually compatible within a larger holocomplex (Figures 4A–4D and S4A–S4E). In MBP purifications from cells co-expressing γ-TuSC$^{Alp6\text{-}MBP}$ and Mto1/2[bonsai], relative levels of copurifying Mto1[bonsai] were ~75% decreased in the absence versus the presence of Mzt1 (Figures 4A, 4B, and S4A). Similarly, in GST and Strep-tag purifications from cells expressing untagged γ-TuSC and either Mto1/2[bonsai]$^{GST\text{-}Mto1[bonsai]}$ or Mto1/2[bonsai]$^{Strep\text{-}Mto1[bonsai]}$, levels of copurifying Alp6 were more than 60%–70% decreased in the absence versus the presence of Mzt1, compared to levels of other proteins (Figures 4C, 4D, and S4B–S4E).

These results suggested that Mzt1 has a specific role in stabilizing Alp6 within the MGM holocomplex, i.e., upon Mto1/2[bonsai] binding to γ-TuSCs. Consistent with this, we found that Mzt1 copurified with Alp6-MBP but not with Alp4-MBP (Figure 4E), confirming previous yeast two-hybrid analysis [11]. CLMS analysis of purified Alp6-MBP:Mzt1 complexes further confirmed that Mzt1 interacts with an N-terminal region of Alp6 (Figure S4F) [11]. These results are consistent with observations for the equivalent proteins in *C. albicans* [10], although they may differ from the overall picture suggested for metazoan cells (see "Concluding Remarks").

We considered two possibilities for how Mzt1 could contribute to stability of Alp6 within the MGM holocomplex. The first possibility was that Mzt1 could bind to both Alp6 and Mto1[bonsai], as a "bridge," as recently described for homologs of these proteins in *C. albicans* [10]. However, Mzt1 did not copurify with GST-tagged Mto1/2[bonsai] unless γ-TuSC was also present (Figure 4F). This suggests that Mzt1 does not bind strongly to Mto1/2[bonsai] on its own, although we cannot rule out that they interact very weakly, or possibly only cooperatively, together with γ-TuSC. Consistent with this result, the amount of Mzt1 copurifying with γ-TuSC$^{Alp6\text{-}MBP}$ was not altered by the presence versus absence of Mto1/2[bonsai] (Figure S4G).

The second possibility, motivated in part by the aberrant aggregation of γ-TuSC when Mzt1 is absent (Figures 1 and S1), was that Mzt1 may support Alp6 stability within the MGM holocomplex more indirectly—for example, by promoting the correct folding or conformation of Alp6. We therefore tested how Mzt1 affects the properties of purified Alp6-MBP alone *in vitro*. In the absence of Mzt1, Alp6-MBP eluted in the void volume during SEC on a Superose 6 column, suggesting that it was forming non-physiological aggregates; by contrast, in the presence of Mzt1, Alp6-MBP co-eluted with Mzt1 as a well-defined species (Figure 4G). This suggests that Mzt1 contributes to folding of a subregion of Alp6 (i.e., the Alp6 N terminus) and/or protects a surface of Alp6 that would otherwise promote aggregation.

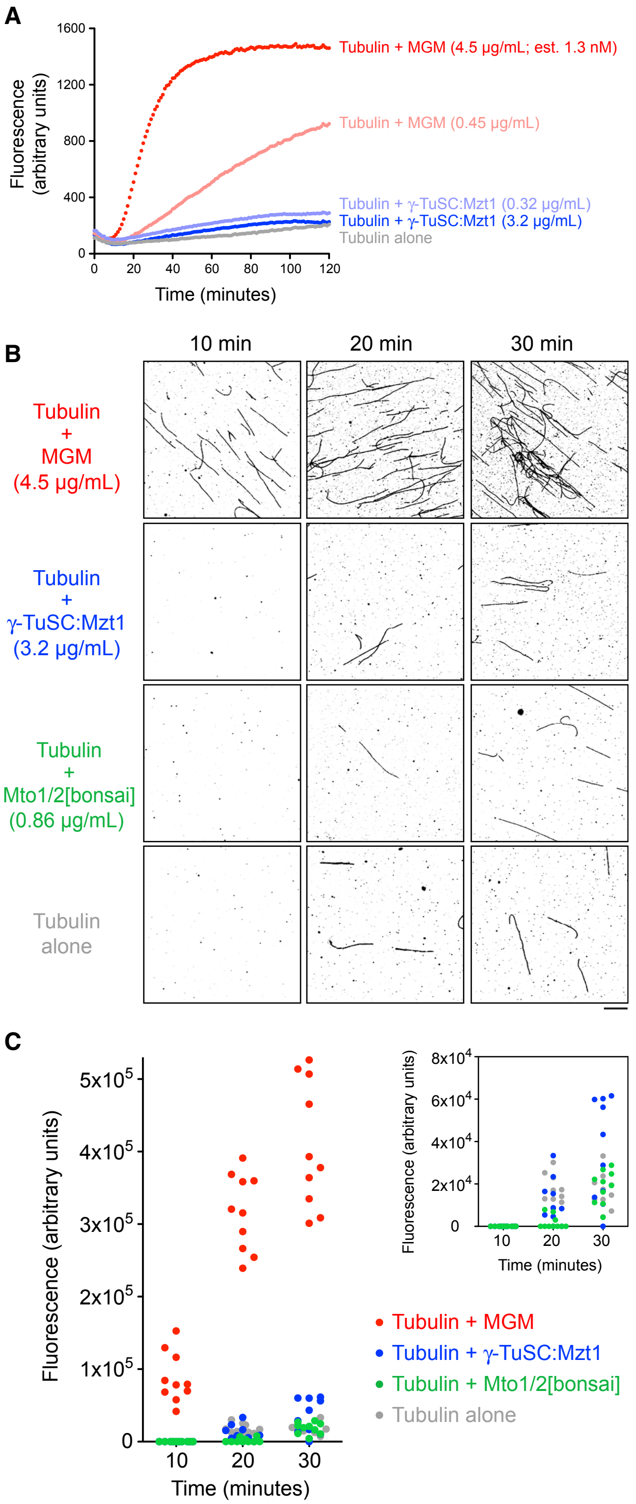

Figure 3. The MGM Holocomplex Is a Potent Microtubule Nucleator *In Vitro*

(A) DAPI fluorescence assay for microtubule (MT) polymerization, using porcine brain tubulin and the indicated complexes and concentrations. The MGM holocomplex nucleates MTs in a dose-dependent manner. The estimated molarity of MGM is based on assumption that Mto1/2[bonsai] and γ-TuSC$^{Alp6\text{-}MBP}$:Mzt1 coassemble into a structure similar to the mammalian γ-TuRC (see STAR Methods). Additional controls are shown in Figures S3A–S3C.

(B) Rhodamine-tubulin fluorescence microscopy assay for MT polymerization.

(C) Quantification of MT polymerization for experiments in (B). Each data point represents total MT fluorescence within a randomly chosen field. Inset shows expanded scale for non-MGM samples. Quantification of MT number per field is shown in Figure S3D. Scale bar, 10 µm.

See also Figure S3.

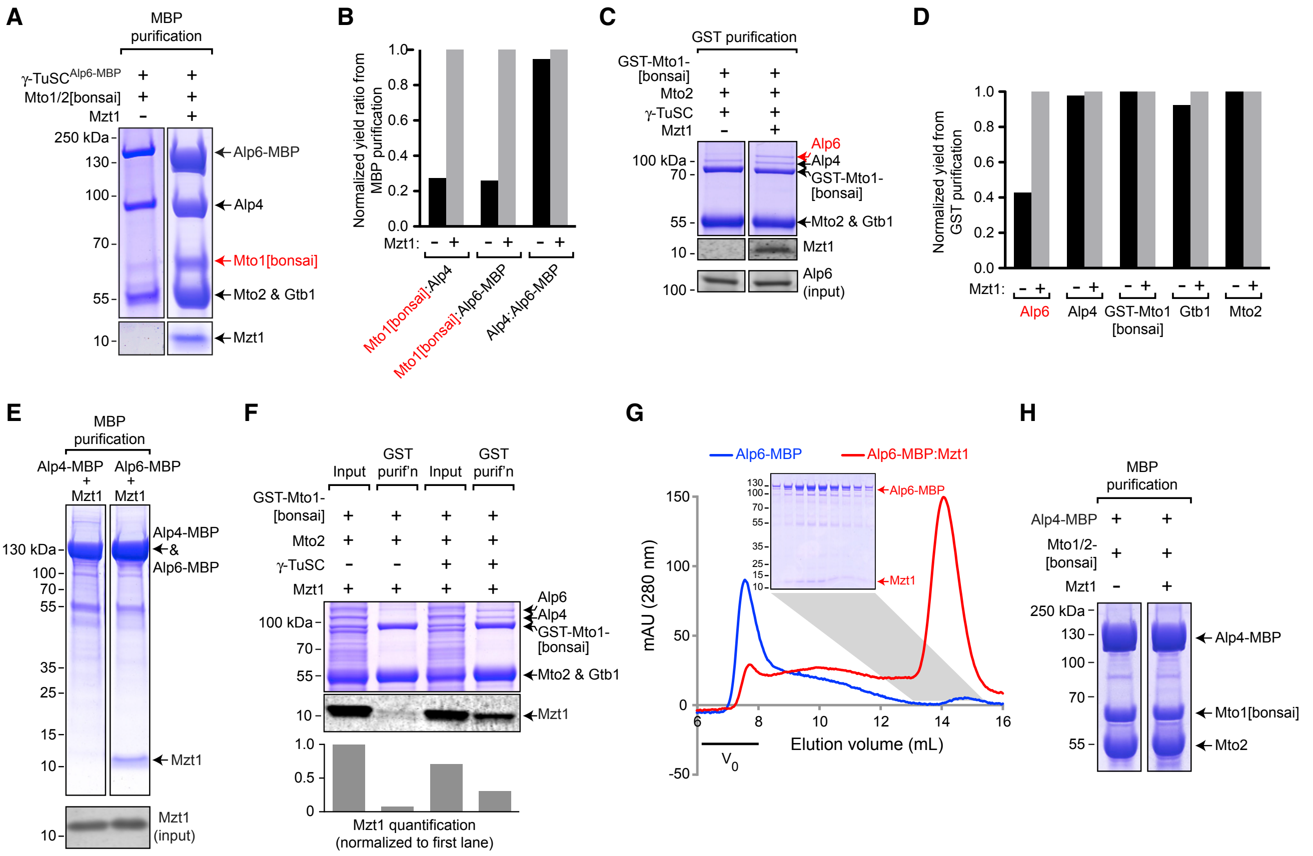

Figure 4. Mzt1 Stabilizes Alp6 in an "Interaction-Competent" State within Higher-Order Complexes

(A) SDS-PAGE of amylose (MBP) purification of coexpressed γ-TuSC$^{Alp6\text{-}MBP}$ and Mto1/2[bonsai] in the absence versus presence of Mzt1. Mzt is required for efficient copurification of Mto1[bonsai] with γ-TuSC. Note that the total yield of γ-TuSC proteins is also decreased. Western blots of protein inputs are shown in Figure S4A.

(B) Quantification of copurification of the indicated proteins from (A). Values for copurification are expressed as ratios, to correct for differences in yield, and normalized to values obtained in the presence of Mzt1.

(C) SDS-PAGE and anti-Mzt1 immunoblot of glutathione-agarose (GST) purification of coexpressed Mto1/2[bonsai]$^{GST\text{-}Mto1[bonsai]}$ and γ-TuSC in the absence versus presence of Mzt1. Mzt is required for efficient copurification of Alp6 with Mto1/2[bonsai]. Anti-Alp6 western blot indicates comparable Alp6 input levels in absence versus presence of Mzt1.

(D) Quantification of copurification of the indicated proteins in (C). Quantification was based on western blots shown in Figure S4B. Equivalent experiments using Mto1/2[bonsai]$^{Strep\text{-}Mto1[bonsai]}$ are shown in Figures S4C–S4E.

(E) SDS-PAGE of amylose (MBP) purification of Alp4-MBP or Alp6-MBP coexpressed with Mzt1. Mzt1 copurifies with Alp6-MBP but not with Alp4-MBP. Anti-Mzt1 western blot indicates comparable Mzt1 levels in the two inputs.

(F) SDS-PAGE and anti-Mzt1 western blot of glutathione-agarose purification of coexpressed Mto1/2[bonsai]$^{GST\text{-}Mto1[bonsai]}$ and Mzt1 in the absence versus presence of γ-TuSC. Mzt1 does not copurify with Mto1/2[bonsai] unless γ-TuSC is also present. The graph shows quantification of anti-Mzt1 western blot, normalized to value in first lane.

(G) Superose 6 size-exclusion chromatography of Alp6-MBP purified alone (blue) or from coexpression with Mzt1 (red). Alp6-MBP elutes in void volume, while Alp6-MBP:Mzt1 elutes much later. Inset shows SDS-PAGE of indicated fractions for Alp6-MBP:Mzt1.

(H) SDS-PAGE of amylose (MBP) purification of coexpressed Alp4-MBP and Mto1/2[bonsai] in the absence versus presence of Mzt1. Mto1/2[bonsai] copurifies with Alp4-MBP, independent of Mzt1 and other γ-TuSC proteins. The control experiment for non-specific binding of Mto1/2[bonsai] to amylose is shown in Figure S4H.

See also Figure S4.

Bringing together our finding that both Alp4 and Alp6 crosslink directly with the Mto1[bonsai] CM1 region (Figure 2C) with our finding that Mzt1 is required specifically to stabilize Alp6 (but not Alp4) within the MGM holocomplex (Figures 4C, 4D, and S4B–S4E), we predicted that Alp4 alone should be able to interact with Mto1/2[bonsai]. Consistent with this, we found that Mto1/2[bonsai] copurified with Alp4-MBP, independent of Alp6, Mzt1, and γ-tubulin (Figures 4H and S4H).

Overall, our results indicate that *S. pombe* Mzt1 maintains Alp6, the homolog of human GCP3, in an "interaction-competent" state that prevents large-scale aggregation of γ-TuSCs and allows Mto1/2[bonsai] to bind to γ-TuSC without disrupting γ-TuSC integrity. This is of particular interest in relation to models motivated by cryo-EM structural analysis of *S. cerevisiae* γ-TuC, which suggests that Spc98 (Alp6/GCP3 homolog) must undergo a conformational change in order

for γ-TuSCs within a potential MT-nucleating complex to form an active nucleator [18, 31, 34] Specifically, rotation of the Spc98 C-terminal half about a central hinge region is thought to allow γ-tubulin molecules from all γ-TuSCs to match the cross-sectional geometry of the MT. Multiple lines of evidence have suggested that CM1 proteins play a critical role in this activation step [10, 17, 18, 23, 35]. In this context, we speculate that in the absence of Mzt1, conformational changes in γ-TuSC induced by Mto1/2[bonsai] binding may lead to Alp6 instability within γ-TuSCs. Conversely, in the presence of Mzt1, Alp6 may be able to tolerate Mto1/2[bonsai]-driven conformational changes without a decrease in affinity for its interactors. Future structural analysis of γ-TuSCs in different states will help to illuminate these issues.

To complement our *in vitro* experiments, we investigated how loss of Mzt1 *in vivo* affects γ-TuSC and its interaction with Mto1/2[bonsai]. We constructed a *3xFLAG-mzt1* allele under control of the thiamine-repressible *nmt81* promoter. In the absence of thiamine, wild-type cells and *nmt81:3xFLAG-mzt1* cells grew equally well (Figure S4I), although further experiments suggested that 3xFLAG-Mzt1 was not fully functional relative to wild-type untagged Mzt1 (Figure S4J). In the presence of thiamine, while wild-type cells grew normally, *nmt81:3xFLAG-mzt1* cells showed almost no growth (Figure S4I). Interestingly, under these repressing conditions, *nmt81:3xFLAG-mzt1* cells showed decreased levels of Alp6 in cell lysates, with a corresponding decrease in coimmunopurification of γ-TuSC proteins with Mto1[bonsai] (Figures S4J and S4K). Overall, these *in vivo* results are consistent with our *in vitro* results indicating a role for Mzt1 in stabilization of Alp6. While the absence of Mzt1 appears to lead to different ultimate fates for Alp6 *in vitro* versus *in vivo* (i.e., aggregation versus probable degradation, respectively), this may be a secondary consequence of different Alp6 concentrations and/or environmental conditions *in vitro* versus *in vivo*.

Mzt1 Is a Dimer in Solution

Mzt1 proteins from *S. pombe*, *C. albicans* and human have been described to form higher-order oligomers *in vitro*, ranging from trimers/tetramers to dodecamers, and this oligomerization is an important feature in several models for Mzt1 function within multimeric γ-TuCs [10, 11, 13, 36]. Given our finding that *S. pombe* Mzt1 has a primary role in maintaining Alp6 stability within the MGM holocomplex, we analyzed the oligomerization state of purified Mzt1 by size-exclusion chromatography with multi-angle light scattering (SEC-MALS; Figures S4L and S4M). While the position of Mzt1 elution suggested a mass of ~40 kDa relative to protein standards, SEC-MALS indicated a molecular mass of ~16.9 kDa. Because *S. pombe* Mzt1 has a theoretical monomeric molecular mass of ~8.8 kDa, this strongly suggests that it is a dimer in solution. While it is possible that Mzt1 has different oligomerization states in different organisms, we note that previous analyses were based on protein expression in *E. coli*. We expressed Mzt1 in insect cells, which may aid proper folding, and we determined molecular mass by SEC-MALS rather than by comparison with elution of protein standards. As Mzt1 is expected to be almost exclusively α-helical [36], it may have an elongated structure, resulting in a disproportionally large Stokes' radius and thus higher apparent mass in conventional SEC. The biological significance of Mzt1 dimerization remains to be investigated.

Concluding Remarks

The reconstituted *S. pombe* MGM holocomplex is a robust MT nucleator *in vitro*: it is active at (estimated) nanomolar/subnanomolar concentrations, and it can efficiently nucleate MTs from mammalian tubulin. MGM may thus be a useful tool for further *in vitro* studies on how nucleators and MT-associated proteins cooperate to regulate MT assembly and dynamics [4, 7].

Importantly, two of our key findings concerning the role of *S. pombe* Mzt1 in MT nucleation differ significantly from recent *in vitro* work involving the equivalent proteins (''*Ca*'' proteins) in the dimorphic yeast *C. albicans* [10]. First, *Ca*Mzt1 has been described as promoting oligomerization of *Ca*γ-TuSC [10]. However, we find that in *S. pombe*, Mzt1 prevents aggregation of γ-TuSC, and this is likely a direct consequence of Mzt1's ability to directly prevent aggregation of Alp6 (Figure 4G). Second, *Ca*Mzt1 interacts not only with *Ca*Spc98 (homolog of *S. pombe* Alp6 and human GCP3; see Figure S1A) but also directly with the CM1 protein *Ca*Spc110, effectively ''bridging'' *Ca*Spc98 and *Ca*Spc110, which also interact independently [10]. The three-way interaction of *Ca*Mzt1, *Ca*Spc98, and *Ca*Spc110 has been suggested to drive *Ca*γ-TuSC:*Ca*Spc110 oligomeric rings into a more compact form, leading to a ~3-fold increase in MT nucleation in the presence versus the absence of *Ca*Mzt1 [10]. By contrast, while we identified interactions between *S. pombe* Mzt1 and Alp6, and between Alp6 and the CM1 protein Mto1[bonsai], we did not find a direct interaction between Mzt1 and Mto1[bonsai] (Figure 4F). Accordingly, our collective results suggest a different view for the role of Mzt1 in MT nucleation in *S. pombe* relative to *C. albicans*, namely that in *S. pombe*, Mzt1 functions to stabilize Alp6 within the γ-TuSC in the face of Mto1-induced conformational changes that may be necessary to generate a functional MT nucleator.

The idea that Mzt1 could have distinct mechanistic roles in different types of MT nucleation complexes may find further support from recent analyses in metazoans. In human, MOZART1 has been shown to bind to the N terminus of not only GCP3 (homolog of Alp6, Spc98, and *Ca*Spc98) but also the related γ-TuRC proteins GCP5 (homolog of *S. pombe* Mod21) and GCP6 (homolog of *S. pombe* Alp16) and also possibly GCP2 (homolog of Alp4, Spc97, and *Ca*Spc97) [10, 13] (Figure S1A). MOZART1 RNAi in human cells in culture profoundly impairs γ-TuRC function, although experiments in different cell types have produced conflicting results as to the specific mechanism involved [8, 10, 13]. In *Drosophila*, Mzt1 interacts only weakly with the GCP3 homolog Grip91, more strongly with the GCP5 homolog Grip128, and not at all with the GCP6 homolog Grip163 [37]. However, in *Drosophila*, Mzt1 is non-essential for viability of the organism and appears to play a critical role only in sperm development [37]. One potential unifying concept from these diverse systems, including the yeasts, is that there may be multiple ways of multimerizing γ-TuSCs to make γ-TuRCs or γ-TuRC-like ring structures in different systems and thus also multiple ways to achieve the conformational changes

necessary to generate an active MT nucleator. These in turn may dictate the specific mechanistic role(s) of Mzt1, as well as its general functional importance.

STAR★METHODS

Detailed methods are provided in the online version of this paper and include the following:

- KEY RESOURCES TABLE
- CONTACT FOR REAGENT AND RESOURCE SHARING
- EXPERIMENTAL MODEL AND SUBJECT DETAILS
  - Bacterial strains
  - Insect cell lines
  - Fission yeast strains
- METHOD DETAILS
  - Plasmids for baculovirus expression
  - Bacmid generation
  - Protein expression in insect cells
  - Protein purification and pulldown assays
  - Density-gradient centrifugation
  - Size-exclusion chromatography and SEC-MALS
  - *In vitro* microtubule nucleation assay
  - Microscopy image analysis
  - Antibody production
  - Crosslinking mass spectrometry
  - Fission yeast strain construction
  - Fission yeast coimmunoprecipitation
  - Electron microscopy
  - Figure preparation
- QUANTIFICATION AND STATISTICAL ANALYSIS
  - Quantification of tubulin polymerization by DAPI fluorescence
  - Quantification of microtubules visualized by fluorescence microscopy
  - Quantification of copurification by western blot and Coomassie Blue staining
- DATA AND SOFTWARE AVAILABILITY

SUPPLEMENTAL INFORMATION

Supplemental Information can be found online at https://doi.org/10.1016/j.cub.2019.05.058.

ACKNOWLEDGMENTS

We thank I. Berger for MultiBac plasmids, H. Thakur for construction of GST-Mto1[bonsai], M. Wear, and M. Nowicki for help with SEC-MALS and plate-reader assays; T. Mitchison, R. Cross, and D. Peet for help/advice on microtubule polymerization; C. Combe for help with mass spectrometry data visualization; D. Kelly for help with fluorescence microscopy; S. Mitchell for help with electron microscopy; and members of our laboratories for discussion. This work was supported by the Wellcome Trust ([094517], [210659] to K.E.S., [108504], [091020] to J.R., Wellcome Trust Multi-User Equipment grant [101527] for the Edinburgh Protein Production Facility, and Wellcome Trust grant [104915] for electron microscopy) and by a Wellcome-University of Edinburgh ISSF Award. E.M.L. was also supported by the Darwin Trust of Edinburgh. The Wellcome Centre for Cell Biology is supported by core funding from the Wellcome Trust [203149].

AUTHOR CONTRIBUTIONS

Conceptualization, S.L.L. and K.E.S.; Methodology, S.L.L., E.M.L., and K.E.S.; Investigation, S.L.L., J.Z., Y.D.T., M.W.T., and K.E.S.; Resources, E.M.L., W.E.B., and J.R.; Writing – Original Draft, S.L.L. and K.E.S.; Writing – Review and Editing, S.L.L. and K.E.S.; Supervision, K.E.S.; Funding Acquisition, K.E.S.

DECLARATION OF INTERESTS

The authors declare no competing interests.

Received: December 18, 2018
Revised: April 29, 2019
Accepted: May 23, 2019
Published: June 20, 2019

REFERENCES

1. Kollman, J.M., Merdes, A., Mourey, L., and Agard, D.A. (2011). Microtubule nucleation by γ-tubulin complexes. Nat. Rev. Mol. Cell Biol. *12*, 709–721.
2. Petry, S., and Vale, R.D. (2015). Microtubule nucleation at the centrosome and beyond. Nat. Cell Biol. *17*, 1089–1093.
3. Lin, T.C., Neuner, A., and Schiebel, E. (2015). Targeting of γ-tubulin complexes to microtubule organizing centers: conservation and divergence. Trends Cell Biol. *25*, 296–307.
4. Roostalu, J., and Surrey, T. (2017). Microtubule nucleation: beyond the template. Nat. Rev. Mol. Cell Biol. *18*, 702–710.
5. Farache, D., Emorine, L., Haren, L., and Merdes, A. (2018). Assembly and regulation of γ-tubulin complexes. Open Biol. *8*, 170266.
6. Paz, J., and Lüders, J. (2018). Microtubule-organizing centers: towards a minimal parts list. Trends Cell Biol. *28*, 176–187.
7. Tovey, C.A., and Conduit, P.T. (2018). Microtubule nucleation by γ-tubulin complexes and beyond. Essays Biochem. *62*, 765–780.
8. Hutchins, J.R., Toyoda, Y., Hegemann, B., Poser, I., Hériché, J.K., Sykora, M.M., Augsburg, M., Hudecz, O., Buschhorn, B.A., Bulkescher, J., et al. (2010). Systematic analysis of human protein complexes identifies chromosome segregation proteins. Science *328*, 593–599.
9. Janski, N., Masoud, K., Batzenschlager, M., Herzog, E., Evrard, J.L., Houlné, G., Bourge, M., Chabouté, M.E., and Schmit, A.C. (2012). The GCP3-interacting proteins GIP1 and GIP2 are required for γ-tubulin complex protein localization, spindle integrity, and chromosomal stability. Plant Cell *24*, 1171–1187.
10. Lin, T.C., Neuner, A., Flemming, D., Liu, P., Chinen, T., Jäkle, U., Arkowitz, R., and Schiebel, E. (2016). MOZART1 and γ-tubulin complex receptors are both required to turn γ-TuSC into an active microtubule nucleation template. J. Cell Biol. *215*, 823–840.
11. Dhani, D.K., Goult, B.T., George, G.M., Rogerson, D.T., Bitton, D.A., Miller, C.J., Schwabe, J.W., and Tanaka, K. (2013). Mzt1/Tam4, a fission yeast MOZART1 homologue, is an essential component of the γ-tubulin complex and directly interacts with GCP3(Alp6). Mol. Biol. Cell *24*, 3337–3349.
12. Masuda, H., Mori, R., Yukawa, M., and Toda, T. (2013). Fission yeast MOZART1/Mzt1 is an essential γ-tubulin complex component required for complex recruitment to the microtubule organizing center, but not its assembly. Mol. Biol. Cell *24*, 2894–2906.
13. Cota, R.R., Teixidó-Travesa, N., Ezquerra, A., Eibes, S., Lacasa, C., Roig, J., and Lüders, J. (2017). MZT1 regulates microtubule nucleation by linking γTuRC assembly to adapter-mediated targeting and activation. J. Cell Sci. *130*, 406–419.
14. Sawin, K.E., Lourenco, P.C., and Snaith, H.A. (2004). Microtubule nucleation at non-spindle pole body microtubule-organizing centers requires fission yeast centrosomin-related protein mod20p. Curr. Biol. *14*, 763–775.

15. Zhang, J., and Megraw, T.L. (2007). Proper recruitment of gamma-tubulin and D-TACC/Msps to embryonic Drosophila centrosomes requires Centrosomin Motif 1. Mol. Biol. Cell *18*, 4037–4049.

16. Samejima, I., Miller, V.J., Groocock, L.M., and Sawin, K.E. (2008). Two distinct regions of Mto1 are required for normal microtubule nucleation and efficient association with the gamma-tubulin complex in vivo. J. Cell Sci. *121*, 3971–3980.

17. Choi, Y.K., Liu, P., Sze, S.K., Dai, C., and Qi, R.Z. (2010). CDK5RAP2 stimulates microtubule nucleation by the gamma-tubulin ring complex. J. Cell Biol. *191*, 1089–1095.

18. Kollman, J.M., Polka, J.K., Zelter, A., Davis, T.N., and Agard, D.A. (2010). Microtubule nucleating gamma-TuSC assembles structures with 13-fold microtubule-like symmetry. Nature *466*, 879–882.

19. Lin, T.C., Neuner, A., Schlosser, Y.T., Scharf, A.N., Weber, L., and Schiebel, E. (2014). Cell-cycle dependent phosphorylation of yeast pericentrin regulates γ-TuSC-mediated microtubule nucleation. eLife *3*, e02208.

20. Venkatram, S., Tasto, J.J., Feoktistova, A., Jennings, J.L., Link, A.J., and Gould, K.L. (2004). Identification and characterization of two novel proteins affecting fission yeast gamma-tubulin complex function. Mol. Biol. Cell *15*, 2287–2301.

21. Samejima, I., Miller, V.J., Rincon, S.A., and Sawin, K.E. (2010). Fission yeast Mto1 regulates diversity of cytoplasmic microtubule organizing centers. Curr. Biol. *20*, 1959–1965.

22. Bao, X.X., Spanos, C., Kojidani, T., Lynch, E.M., Rappsilber, J., Hiraoka, Y., Haraguchi, T., and Sawin, K.E. (2018). Exportin Crm1 is repurposed as a docking protein to generate microtubule organizing centers at the nuclear pore. eLife *7*, e33465.

23. Lynch, E.M., Groocock, L.M., Borek, W.E., and Sawin, K.E. (2014). Activation of the γ-tubulin complex by the Mto1/2 complex. Curr. Biol. *24*, 896–903.

24. Vardy, L., and Toda, T. (2000). The fission yeast gamma-tubulin complex is required in G(1) phase and is a component of the spindle assembly checkpoint. EMBO J. *19*, 6098–6111.

25. Vinh, D.B., Kern, J.W., Hancock, W.O., Howard, J., and Davis, T.N. (2002). Reconstitution and characterization of budding yeast gamma-tubulin complex. Mol. Biol. Cell *13*, 1144–1157.

26. Oegema, K., Wiese, C., Martin, O.C., Milligan, R.A., Iwamatsu, A., Mitchison, T.J., and Zheng, Y. (1999). Characterization of two related Drosophila gamma-tubulin complexes that differ in their ability to nucleate microtubules. J. Cell Biol. *144*, 721–733.

27. Gunawardane, R.N., Martin, O.C., Cao, K., Zhang, L., Dej, K., Iwamatsu, A., and Zheng, Y. (2000). Characterization and reconstitution of Drosophila gamma-tubulin ring complex subunits. J. Cell Biol. *151*, 1513–1524.

28. Zheng, Y., Wong, M.L., Alberts, B., and Mitchison, T. (1995). Nucleation of microtubule assembly by a gamma-tubulin-containing ring complex. Nature *378*, 578–583.

29. Moritz, M., Braunfeld, M.B., Guénebaut, V., Heuser, J., and Agard, D.A. (2000). Structure of the gamma-tubulin ring complex: a template for microtubule nucleation. Nat. Cell Biol. *2*, 365–370.

30. Bonne, D., Heuséle, C., Simon, C., and Pantaloni, D. (1985). 4′,6-Diamidino-2-phenylindole, a fluorescent probe for tubulin and microtubules. J. Biol. Chem. *260*, 2819–2825.

31. Kollman, J.M., Greenberg, C.H., Li, S., Moritz, M., Zelter, A., Fong, K.K., Fernandez, J.J., Sali, A., Kilmartin, J., Davis, T.N., and Agard, D.A. (2015). Ring closure activates yeast γTuRC for species-specific microtubule nucleation. Nat. Struct. Mol. Biol. *22*, 132–137.

32. Anders, A., Lourenço, P.C., and Sawin, K.E. (2006). Noncore components of the fission yeast gamma-tubulin complex. Mol. Biol. Cell *17*, 5075–5093.

33. Masuda, H., and Toda, T. (2016). Synergistic role of fission yeast Alp16GCP6 and Mzt1MOZART1 in γ-tubulin complex recruitment to mitotic spindle pole bodies and spindle assembly. Mol. Biol. Cell *27*, 1753–1763.

34. Kollman, J.M., Zelter, A., Muller, E.G., Fox, B., Rice, L.M., Davis, T.N., and Agard, D.A. (2008). The structure of the gamma-tubulin small complex: implications of its architecture and flexibility for microtubule nucleation. Mol. Biol. Cell *19*, 207–215.

35. Muroyama, A., Seldin, L., and Lechler, T. (2016). Divergent regulation of functionally distinct γ-tubulin complexes during differentiation. J. Cell Biol. *213*, 679–692.

36. Cukier, C.D., Tourdes, A., El-Mazouni, D., Guillet, V., Nomme, J., Mourey, L., Milon, A., Merdes, A., and Gervais, V. (2017). NMR secondary structure and interactions of recombinant human MOZART1 protein, a component of the gamma-tubulin complex. Protein Sci. *26*, 2240–2248.

37. Tovey, C.A., Tubman, C.E., Hamrud, E., Zhu, Z., Dyas, A.E., Butterfield, A.N., Fyfe, A., Johnson, E., and Conduit, P.T. (2018). gamma-TuRC Heterogeneity Revealed by Analysis of Mozart1. Curr. Biol *28*, 2314–2323.e2316.

38. Trowitzsch, S., Bieniossek, C., Nie, Y., Garzoni, F., and Berger, I. (2010). New baculovirus expression tools for recombinant protein complex production. J. Struct. Biol. *172*, 45–54.

39. Berger, I., Fitzgerald, D.J., and Richmond, T.J. (2004). Baculovirus expression system for heterologous multiprotein complexes. Nat. Biotechnol. *22*, 1583–1587.

40. Busso, D., Delagoutte-Busso, B., and Moras, D. (2005). Construction of a set Gateway-based destination vectors for high-throughput cloning and expression screening in Escherichia coli. Anal. Biochem. *343*, 313–321.

41. Noguchi, C., Garabedian, M.V., Malik, M., and Noguchi, E. (2008). A vector system for genomic FLAG epitope-tagging in Schizosaccharomyces pombe. Biotechnol. J. *3*, 1280–1285.

42. Ye, Y., Lee, I.J., Runge, K.W., and Wu, J.Q. (2012). Roles of putative Rho-GEF Gef2 in division-site positioning and contractile-ring function in fission yeast cytokinesis. Mol. Biol. Cell *23*, 1181–1195.

43. Petersen, J., and Russell, P. (2016). Growth and the environment of Schizosaccharomyces pombe. Cold Spring Harb. Protoc. https://doi.org/10.1101/pdb.top079764.

44. Bieniossek, C., Richmond, T.J., and Berger, I. (2008). MultiBac: multigene baculovirus-based eukaryotic protein complex production. Curr. Protoc. Protein Sci. *51*, 5.20.1–5.20.26.

45. Samejima, I., Lourenço, P.C., Snaith, H.A., and Sawin, K.E. (2005). Fission yeast mto2p regulates microtubule nucleation by the centrosomin-related protein mto1p. Mol. Biol. Cell *16*, 3040–3051.

46. Shevchenko, A., Wilm, M., Vorm, O., and Mann, M. (1996). Mass spectrometric sequencing of proteins silver-stained polyacrylamide gels. Anal. Chem. *68*, 850–858.

47. Ishihama, Y., Rappsilber, J., and Mann, M. (2006). Modular stop and go extraction tips with stacked disks for parallel and multidimensional Peptide fractionation in proteomics. J. Proteome Res. *5*, 988–994.

48. Rappsilber, J., Mann, M., and Ishihama, Y. (2007). Protocol for micro-purification, enrichment, pre-fractionation and storage of peptides for proteomics using StageTips. Nat. Protoc. *2*, 1896–1906.

49. Rappsilber, J., Ishihama, Y., and Mann, M. (2003). Stop and go extraction tips for matrix-assisted laser desorption/ionization, nanoelectrospray, and LC/MS sample pretreatment in proteomics. Anal. Chem. *75*, 663–670.

50. Cox, J., and Mann, M. (2008). MaxQuant enables high peptide identification rates, individualized p.p.b.-range mass accuracies and proteome-wide protein quantification. Nat. Biotechnol. *26*, 1367–1372.

51. Lock, A., Rutherford, K., Harris, M.A., Hayles, J., Oliver, S.G., Bähler, J., and Wood, V. (2019). PomBase 2018: user-driven reimplementation of the fission yeast database provides rapid and intuitive access to diverse, interconnected information. Nucleic Acids Res. *47* (D1), D821–D827.

52. Fischer, L., and Rappsilber, J. (2017). Quirks of error estimation in cross-linking/mass spectrometry. Anal. Chem. *89*, 3829–3833.

53. Basi, G., Schmid, E., and Maundrell, K. (1993). TATA box mutations in the Schizosaccharomyces pombe nmt1 promoter affect transcription efficiency but not the transcription start point or thiamine repressibility. Gene *123*, 131–136.

54. Ekwall, K., and Thon, G. (2017). Genetic analysis of Schizosaccharomyces pombe. Cold Spring Harb. Protoc. https://doi.org/10.1101/pdb.top079772.

55. Borek, W.E., Groocock, L.M., Samejima, I., Zou, J., de Lima Alves, F., Rappsilber, J., and Sawin, K.E. (2015). Mto2 multisite phosphorylation inactivates non-spindle microtubule nucleation complexes during mitosis. Nat. Commun. *6*, 7929.

56. Perez-Riverol, Y., Csordas, A., Bai, J., Bernal-Llinares, M., Hewapathirana, S., Kundu, D.J., Inuganti, A., Griss, J., Mayer, G., Eisenacher, M., et al. (2019). The PRIDE database and related tools and resources in 2019: improving support for quantification data. Nucleic Acids Res. *47* (D1), D442–D450.

STAR★METHODS

KEY RESOURCES TABLE

REAGENT or RESOURCE	SOURCE	IDENTIFIER
Antibodies		
Sheep anti-Mzt1 (serum)	This paper	N/A
Sheep anti-Alp6 (serum)	Homemade lab stock	N/A
Sheep anti-Alp4 (serum)	Homemade lab stock	N/A
Sheep anti-Alp6, affinity-purified	Homemade lab stock	N/A
Sheep anti-Alp4, affinity-purified	Homemade lab stock	N/A
Mouse monoclonal anti-γ-tubulin antibody, clone GTU-88	Sigma-Aldrich	Cat# T6557; RRID:AB_477584
Sheep anti-Mto1 (serum)	Homemade lab stock	N/A
Sheep anti-Mto2 (serum)	Homemade lab stock	N/A
Sheep anti-GFP, affinity-purified	Homemade lab stock	N/A
Mouse monoclonal Anti-FLAG® M2 antibody	Sigma-Aldrich	Cat# F1804; RRID:AB_262044
Mouse monoclonal anti-Goat/Sheep IgG antibody, clone GT-34	Sigma-Aldrich	Cat# G2904; RRID:AB_259856
IRDye 800CW Donkey anti-Mouse IgG (H+L)	LI-COR	Cat# 926-32212; RRID:AB_621847
Bacterial and Virus Strains		
Escherichia coli DH10 EMBacY	Imre Berger Lab [38];	N/A
Escherichia coli BL21-CodonPlus (DE3)-RIL	Agilent	Cat# 230245
Chemicals, Peptides, and Recombinant Proteins		
4,6-diamidino-2-phenylindole, dihydrochloride (DAPI)	Thermo Fisher Scientific	Cat# D1306
Tubulin (porcine) > 99%	Cytoskeleton, Inc.	Cat# T240
Rhodamine-labeled porcine tubulin	Cytoskeleton, Inc.	Cat# TL590M
Chymostatin	Melford	Cat# C1104
Leupeptin hydrochloride > 90%	Apollo Scientific	Cat# BIMI2442
Aprotinin	Apollo Scientific	Cat# BIMI2132
Antipain dihydrochloride	Apollo Scientific	Cat# BIA0201
Pepstatin A	Apollo Scientific	Cat# BIMI2205
E-64	Apollo Scientific	Cat# BIMI2157
Amylose resin	New England Biolabs	Cat# E8021L
Glutathione-agarose resin	Sigma-Aldrich	Cat# G4510
Strep-Tactin Sepharose resin	IBA GmBH	Cat# 2-1201-010
Fractogel EMD Chelate	Merck	Cat# 1.10338.0010
X-tremeGENE HP DNA Transfection Reagent	Roche	Cat# 6366244001
GIBCO Express Five SFM media	Thermo Fisher Scientific	Cat# 10486-025
Sf-900 II SFM	Thermo Fisher Scientific	Cat# 10902-096
L-Glutamine 200mM	Thermo Fisher Scientific	Cat# 25030024
Cre recombinase	New England Biolabs	Cat# M0298
Dextran sulfate sodium salt	Sigma-Aldrich	Cat# 31404
SYPRO Ruby gel stain	Lonza	Cat# LZ50562
1-ethyl-3-(3-dimethylaminopropyl)carbodiimide hydrochloride (EDC)	Thermo Fisher Scientific	Cat# 22980
sulfosuccinimidyl 4,4'-azipentanoate (Sulfo-NHS-Diazirine) (Sulfo-SDA)	Thermo Fisher Scientific	Cat# 26173
N-hydroxysulfosuccinimide (Sulfo-NHS)	Thermo Fisher Scientific	Cat# 24525
Ammonium bicarbonate	Sigma-Aldrich	Cat# 09830
1,4-Dithiothreitol (DTT)	Sigma-Aldrich	Cat# 10197777001

(*Continued on next page*)

Continued

REAGENT or RESOURCE	SOURCE	IDENTIFIER
Iodoacetamide	Sigma-Aldrich	Cat# I1149
Pierce™ Trypsin protease	Thermo Fisher Scientific	Cat# 90057
RunBlue™ SDS Running Buffer	Expedeon	Cat# NXB50500
Protein G Dynabeads	Thermo Fisher Scientific	Cat# 10003D
Bacto Yeast Extract	BD Biosciences	Cat# 212750
Bacto Agar	BD Biosciences	Cat# 214030
Critical Commercial Assays		
Gateway® LR Clonase® II enzyme mix	Thermo Fisher Scientific	Cat# 11791-020
Gateway® BP Clonase® II enzyme mix	Thermo Fisher Scientific	Cat# 11789-020
Deposited Data		
MS proteomics data deposited to ProteomeXchange Consortium via PRIDE	This paper	ProteomeXchange: PXD012624
Experimental Models: Cell Lines		
Sf9 cells	Thermo Fisher Scientific	Cat# 11496015
High Five (BTI-TN-5B1-4) cells	Thermo Fisher Scientific	Cat# B85502
Experimental Models: Organisms/Strains		
Schizosaccharomyces pombe	This paper	NCBI:txid4896
h- ade6-M210 leu1-32 ura4-D18	This paper	KS516
h- hphMX6:nmt81:3xFLAG-mzt1 ade6-M210 leu1-32 ura4-D18	This paper	KS7623
h- mto1(131-549)-mEcitrine:kanMX6 natMX6:Z:adh15:mCherry-Atb2 ade6-M210 ura4-D18 leu1-32	This paper	KS10055
h- hphMX6:nmt81:3xFLAG-mzt1 mto1(131-549)-mEcitrine:kanMX6 natMX6:Z:adh15:mCherry-Atb2 ade6-M210 ura4-D18 leu1-32	This paper	KS10059
Oligonucleotides		
Primers for PCR, see Table S1	This paper	N/A
Recombinant DNA		
pFL vector	Imre Berger Lab [38];	pKS1219
pUCDM vector	Imre Berger Lab [39];	pKS1251
pMK-RQ XhoI_L21_Gtb1_NheI *(to generate untagged Gtb1)*	GeneArt (this paper)	pKS1815
pMA BamHI_Alp4_TAA_PstI *(to generate untagged Alp4)*	GeneArt (this paper)	pKS1807
pMA BamHI_L21_Alp6_TAA_PstI *(to generate untagged Alp6)*	GeneArt (this paper)	pKS1812
pMK-RQ KasI_MBP_His_PstI *(to generate C-terminal MBP-6xHis tags)*	GeneArt (this paper)	pKS1811
pMK-RQ BamHI_Alp4_KasI *(to generate tagged Alp4)*	GeneArt (this paper)	pKS1814
pMK-RQ BamHI_L21_Alp6_KasI *(to generate tagged Alp6)*	GeneArt (this paper)	pKS1810
pMA-T Mzt1 (insect cells) *(to generate Mzt1 codon-optimized for insect cell expression)*	GeneArt (this paper)	pKS1813
pFL_Alp6-MBP_Gtb1	This paper	pKS1791
pUCDM_Alp4_Gtb1	This paper	pKS1788
γ-TuSC Alp6-MBP	This paper	pKS1794
pFL_Alp4_Gtb1	This paper	pKS1804
pFL_Mzt1-6xHis	This paper	pKS1801
pFL_6xHis-Mto1/2[bonsai]	This paper	pKS1225
pFL_GST-Mto1bonsai	This paper	pKS1548
pFL_6xHis-Mto2	This paper	pKS1258
pUCDM_Alp6_Gtb1	This paper	pKS1785
γ-TuSC Untagged	This paper	pKS1805
pFL_Alp4-MBP	This paper	pKS1795
pFL_Alp6-MBP	This paper	pKS1796
pFL_Strep-Mto1/2[bonsai]	This paper	pKS1799

(*Continued on next page*)

REAGENT or RESOURCE	SOURCE	IDENTIFIER
Continued		
p0GWA vector	Didier Busso Lab [40];	pKS537
pGGWA vector	Didier Busso Lab [40];	pKS538
pMA-T Mzt1_bac *(to generate Mzt1 codon-optimized for bacterial expression)*	GeneArt (this paper)	pKS1809
p0GWA Mzt1_6xHis	This paper	pKS1821
pGGWA GST_Mzt1_6xHis	This paper	pKS1822
pFA6a-hphMX6-P81nmt1-3FLAG	Addgene [41];	RRID:Addgene_19350; pKS1284
pFA6a-mEcitrine-KanMX6	Addgene [42];	RRID:Addgene_105150; pKS1330
Software and Algorithms		
ImageJ (Fiji)	NIH	RRID:SCR_002285
GraphPad Prism 7 software	GraphPad	RRID:SCR_002798
S. pombe database (released in August, 2013)	PomBase	RRID:SCR_006586
Image Studio Lite	LI-COR	RRID:SCR_013715
Image Lab™	Bio-Rad	RRID:SCR_014210
Other		
Bolt® 4-12% Bis-Tris Gel	Thermo Fisher Scientific	Cat# NW04125BOX; Cat# NW04127BOX; Cat# NW04122BOX; Cat# NW04120BOX
RunBlue™ 4-20% Teo-Tricine SDS Gels	Expedeon	Cat# NXG42012
ÄKTA chromatography system	GE Healthcare	N/A
ÄKTAmicro chromatography system	GE Healthcare	N/A
Viscotek SEC-MALS 20 detector	Malvern Instruments	N/A
Viscotek RI detector VE3580	Malvern Instruments	N/A
Amicon Ultra-4 Ultracel-3K centrifugal filter unit	Millipore	Cat# UFC800324
Amicon Ultra-15 Ultracel-3K centrifugal filter unit	Millipore	Cat# UFC900324
Half area 96 well plate	Greiner Bio-One Inc.	Cat# 675076
Coverslips 24x50 mm No. 1.5	VWR	Cat# 631-0147
Gradient Master 107ip	Biocomp Instruments	N/A
SpectraMax M5 multi-mode microplate reader	Molecular Devices	N/A
Superose 6 10/300	GE Healthcare	Cat# 17-5172-01
Superdex 75 10/300	GE Healthcare	Cat# 17-5174-01
Beckman Coulter Optima MAX Ultracentrifuge	Beckman Coulter	N/A
ChemiDoc™ Imager	Bio-Rad	Cat# 17001401
Odyssey CLx fluorescence imager	LI-COR	N/A
Airyscan confocal microscope	Zeiss	LSM880
Freezer/mill® LLC 6870	SPEX SamplePrep	N/A
400 mesh copper grid with carbon film support	TAAB	Cat# C169/100
Filter paper, grade 1	Whatman	Cat# 1001-090
JEM-1400 transmission electron microscope	JEOL	JEM-1400
OneView 4k x 4k CMOS detector	Gatan	N/A

CONTACT FOR REAGENT AND RESOURCE SHARING

Further requests for reagents and resource sharing should be directed to and will be fulfilled by the Lead Contact, Prof. Kenneth Sawin (ken.sawin@ed.ac.uk).

EXPERIMENTAL MODEL AND SUBJECT DETAILS

Bacterial strains

Escherichia coli strains DH5alpha, DH10 EMBacY [38] (kindly provided by Imre Berger, University of Bristol, UK) and BL21-CodonPlus (DE3)-RIL (Agilent) were grown at 37°C on LB agar plates or in LB liquid medium with shaking at 200 rpm. For experiments

involving protein expression in BL21-CodonPlus (DE3)-RIL cells, cultures were grown at 18°C prior to and during induction of expression.

Insect cell lines

Spodoptera frugiperda Sf9 cells (Thermo Fisher Scientific) and *Trichoplusia ni* BTI-TN-5B1-4 High Five cells (Thermo Fisher Scientific) were maintained as both adherent and suspension cultures at 27°C. Sf9 cells were grown in Sf-900 II SFM medium (Thermo Fisher Scientific), and High Five cells were grown in Express Five SFM medium with supplemented L-glutamine (Thermo Fisher Scientific). Adherent cultures were split 1:4 when confluent (every 2-3 days). Suspension cultures were shaken in flasks at 120 rpm and maintained at ~2×10^6 cells/mL to ensure optimal growth conditions. High Five suspension cultures were supplemented with 25 μg/mL dextran sulfate (Sigma-Aldrich) to prevent cell clumping.

Fission yeast strains

Fission yeast *Schizosaccharomyces pombe* cells were grown either in YE5S rich medium, using Bacto Yeast Extract (BD Biosciences) or in EMMG (also known as PMG) minimal medium [43]. Cells were grown on plates containing 2% Bacto Agar (BD Biosciences) or in liquid culture with shaking at 150-160 rpm, at either 25°C or 32°C, depending on requirements. Media supplements (e.g., adenine, leucine, and uracil) were used at 175 mg/L. For experiments shown in Figure S4, cells were grown in EMMG medium at 32°C.

METHOD DETAILS

Plasmids for baculovirus expression

Recombinant proteins were produced using the MultiBac baculovirus system, and plasmids were constructed using MultiBac transfer vectors pFL and pUCDM, kindly provided by Imre Berger [38, 39]. Coding sequences for Alp4 (pKS1807, pKS1814), Alp6 (pKS1810, pKS1812), Gtb1 (pKS1815), Mzt1 (pKS1813), and the maltose-binding protein (MBP) tag (pKS1811) were codon-optimized for insect-cell expression and synthesized by GeneArt (Thermo Fisher Scientific). In order to construct a modular expression system, coding sequences were flanked by specific restriction sites to allow subcloning into pFL and pUCDM, each of which contain two separate multicloning sites (MCSs), each under control of a different baculovirus promoter [38, 39].

A plasmid for expression of γ-TuSC$^{\text{Alp6-MBP}}$, pKS1794, was constructed by a multistep process, as follows. Alp6 plus MBP coding sequences (from pKS1810 and pKS1811, respectively) were subcloned into BamHI/PstI sites within MCS2 of pFL, to generate pKS1796, which allows expression of Alp6-MBP under control of the polh promoter. The Gtb1 coding sequence from pKS1815 was then subcloned into XhoI/NheI sites within MCS1, to generate pKS1791, which further allows expression of Gtb1 under control of the p10 promoter. In parallel, the Alp4 (untagged) coding sequence from pKS1807 and the Gtb1 coding sequence from pKS1815 were subcloned into pUCDM, using BamHI/PstI sites within MCS2 and XhoI/NheI sites within MCS1, respectively. This generated pKS1788, which allows expression of untagged Alp4 and Gtb1 under control of polh and p10 promoters, respectively. pKS1791 and pKS1788 were then combined by site-specific recombination, using Cre recombinase (New England Biolabs), to generate pKS1794.

A plasmid for expression of untagged γ-TuSC, pKS1805, was constructed by similar methods. Alp4 and Gtb1 coding sequences (from pKS1807 and pKS1815, respectively) were subcloned stepwise as above into pFL, to generate pKS1804, which allows expression of untagged Alp4 and Gtb1 under control of polh and p10 promoters, respectively. In parallel, Alp6 (untagged) and Gtb1 coding sequences (from pKS1812 and pKS1815, respectively) were subcloned stepwise as above into pUCDM, to generate pKS1785, which allows expression of untagged Alp6 and Gtb1 under control of polh and p10 promoters, respectively. pKS1804 and pKS1785 were then combined by site-specific recombination, using Cre recombinase, to generate pKS1805.

A plasmid for expression of Alp4-MBP, pKS1795, was constructed by subcloning Alp4 plus MBP coding sequences (from pKS1814 and pKS1811, respectively) into BamHI/PstI sites within MCS2 of pFL, allowing expression of Alp4-MBP under control of the polh promoter.

A plasmid for expression of Mzt1-6xHis, pKS1801, was constructed as follows. To introduce a hexahistidine (6xHis) tag on the C terminus of Mzt1, the Mzt1 coding sequence was amplified from pKS1813 by PCR, using primers OKS2838 and OKS2839. The resulting product was cloned into BamHI/PstI sites within MCS2 of pFL, allowing expression of Mzt1-6xHis under control of the polh promoter.

A plasmid for expression of 6xHis-Mto1/2[bonsai], pKS1225, was constructed by stepwise cloning of a PCR product for 6xHis-Mto1[bonsai] (comprising amino acids 131-549 of Mto1) into NcoI/NsiI sites within MCS1 of pFL and a PCR product for Mto2 into BamHI/SalI sites within MCS2, allowing expression of 6xHis-Mto1[bonsai] and Mto2 under control of the p10 and polh promoters, respectively. The 6xHis-Mto1[bonsai] coding sequence was amplified from pKS272 (lab stock), using primers OKS2182 and OKS2184. The Mto2 coding sequence was amplified from pKS415 (lab stock), using primers OKS2185 and OKS2186. The Mto2 PCR product contained a BclI site (compatible with BamHI) rather than a BamHI site, because of internal BamHI sites within the Mto2 coding sequence. A similar approach was used to construct a separate plasmid for single expression of 6xHis-Mto2, pKS1258. The Mto2 coding sequence was amplified from pKS415, using primers OKS2320 and OKS2186, and the PCR product was cloned into BamHI/SalI sites within MCS2 of pFL, allowing expression of 6xHis-Mto2 under control of the polh promoter.

A plasmid for expression of GST-Mto1[bonsai], pKS1548, was constructed by subcloning the GST coding sequence into XmaI/XhoI sites upstream of (and in frame with) the 6xHis-Mto1[bonsai] sequence that had already been cloned into MCS1 of pFL as described above (i.e., without Mto2).

A plasmid for expression of Strep-Mto1/2[bonsai], pKS1799, was constructed by inserting a Twin-Strep-tag® sequence on the N terminus of Mto1[bonsai] via sequential PCR using the forward primers OKS2884 and OKS2883 and reverse primer OKS2978. For simplicity, we refer to this as a Strep tag. The Strep-Mto1[bonsai] PCR product was cloned into XhoI/SphI sites within MCS1 of pFL, allowing expression of Strep-Mto1[bonsai] under control of the p10 promoter. Mto2 was cloned into MCS2, as described above.

Bacmid generation

Bacmids were generated by Tn7-dependent transposition of the pFL-based and pFL/pUCDM/recombination-based plasmids described above into DH10 EMBacY *E. coli* (containing the EMBacY baculovirus genome). Transformed cells were plated on agar plates containing 50μg/mL kanamycin, 10μg/mL tetracycline, 7 μg/mL gentamycin, 0.2 mM IPTG and 200 μg/mL 5-bromo-3-indolyl-beta-galactoside (Bluo-Gal) and incubated at 37°C for 48 hours until blue/white colonies were formed. 'Positive' white colonies (indicating integration) were identified, and single colonies were inoculated into 5 mL of LB containing 50μg/mL kanamycin, 10 μg/mL tetracycline and 7 μg/mL gentamycin and incubated overnight at 37°C with shaking at 200 rpm. Recombinant bacmid DNA was isolated for insect cell transfection.

Protein expression in insect cells

For baculovirus production, Sf9 cells were used, and for protein expression, High Five cells were used. For the initial production of virus (V_0), Sf9 cells were infected with EMBacY bacmid containing the gene(s) of interest. First, bacmid DNA was isolated by alkaline lysis followed by precipitation. DNA was then resuspended in 40 μL of ddH_2O and combined with 400 μL Sf-900 II SFM and 20 μL X-tremeGENE HP DNA transfection reagent (Roche) and incubated for 30 min at room temperature. 100 μL of this mixture was then added to each of four wells seeded with 0.6×10^6 Sf9 cells in a 6-well tissue culture plate, followed by incubation at 27°C. After 48-60 hours, V_0 was harvested by collecting the supernatant and filtering through a 0.45 μm syringe filter.

Amplification of viruses (V_1 and V_2) was performed as previously described [44]. Briefly, Sf9 cells were grown to 2×10^6 cells/mL and then infected with V_0 or V_1 at a 1:10 (v/v) ratio relative to cell-culture medium. After 48 hours, V_1 and V_2 were collected by gentle centrifugation of the culture at 500 x *g* (Heraeus Megafuge) for 10 min, followed by recovery of the supernatant, which was then filtered through a 0.45 μm syringe filter. The amount of each virus used was optimized for protein expression. V_0 and V_1 were stored at 4°C. For coexpression of multiprotein complexes, High Five cells were grown to 2×10^6 cells/mL prior to infection with V_2 baculovirus. V_2 baculovirus was used at an equal volume for each protein, at a V_2-to-High Five-culture ratio of 1:80 (v/v). YFP fluorescence was monitored as an indication of protein expression, and cells were harvested at peak fluorescence (48-72 hours post-infection). To harvest, cells were centrifuged at 1000 x *g* for 15 min, and cell pellets were washed with phosphate-buffered saline (PBS), repelleted and snap-frozen in liquid nitrogen for storage at −80°C.

For expression of γ-TuSC$^{Alp6\text{-}MBP}$, γ-TuSC$^{Alp6\text{-}MBP}$:Mzt1 and MGM holocomplex, cells were coinfected with equal volumes of V_2 for γ-TuSC$^{Alp6\text{-}MBP}$, Mto1/2[bonsai] and Mzt1 viruses, together with an equal volume of V_2 virus for expression of Alp4 and Gtb1. This additional virus was used to ensure that expression of untagged proteins was in excess (i.e., not limiting). This was confirmed from western blot analyses of unbound proteins after overnight binding to beads for affinity purification.

Protein purification and pulldown assays

Purification of γ-TuSC$^{Alp6\text{-}MBP}$, γ-TuSC$^{Alp6\text{-}MBP}$:Mzt1 and the MGM holocomplex was achieved via affinity chromatography using the MBP tag on Alp6. Briefly, insect cell pellets were resuspended and lysed in ten volumes of cold HB100 buffer (40 mM K-HEPES, 100 mM KCl, 1 mM EGTA, 1 mM $MgCl_2$, 0.1 mM GTP, 1 mM DTT pH7.5) with 1 mM PMSF and 10 μg/mL each of CLAAPE protease inhibitors (chymostatin, leupeptin, aprotinin, antipain, pepstatin, E-64). Lysates were sonicated three times for 15 s using a tip sonicator, with a 5-min interval on ice between sonications. Sonicated lysates were then clarified by centrifugation, first at 4000 x *g* (Megafuge, Thermo Scientific Heraeus) for 30 min at 4°C, and then at 90,000 rpm for 10 min at 4°C (Optima MAX Ultracentrifuge; Beckman Coulter). Clarified cell lysates were then incubated with amylose resin (New England Biolabs) overnight at 4°C on a tube roller. On the following day, beads with bound proteins were washed sequentially with a minimum of 40 column volumes of HB100 buffer and eluted with 50 mM maltose in HB100 at 4°C. Purity of samples were analyzed by SDS-PAGE and Coomassie Blue staining. The MBP tag was generally not removed because, due to the multiple copies of Alp6 in the multicopy protein complexes, efficient elution of the complexes would require essentially 100% cleavage of the tag. In addition, as described further below, complexes were found to be similarly functional both with and without removal of the tag.

Mzt1 was purified via a 6xHis tag on the Mzt1 C terminus. Clarified cell lysates were loaded on a Ni^{2+}-charged 1 mL HiTrap IMAC column (GE Healthcare) and eluted using a 5%–50% gradient with Buffer A = 100 mM Tris, 150 mM NaCl pH7.5 and Buffer B = 100 mM Tris, 150 mM NaCl, 1 M imidazole pH 7.5. Fractions corresponding to the protein peak were collected, concentrated on an Amicon Ultra-4 or Ultra-15 centrifugal filter unit (3 kDa cut-off; Millipore) and loaded onto a Superdex 75 10/300 column attached to an ÄKTA chromatography system (GE Healthcare). The column buffer was 100 mM Tris, 150 mM NaCl pH 7.5. Protein purity was confirmed by SDS-PAGE and Coomassie Blue staining. For the analysis of Mzt1 shown in Figure S4H, fractions were run on a RunBlue 4%–20% Teo-Tricine SDS gel (Expedeon) with RunBlue SDS Running Buffer (Expedeon). On this gel system, Mzt1 migrates with a slightly lower apparent molecular weight compared to the gel systems used in other figures.

Mto1/2[bonsai] was purified via a 6xHis tag on the N terminus of Mto1[bonsai]. Clarified cell lysates were precipitated with ammonium sulfate at 20% saturation by incubating with an appropriate amount of ammonium sulfate (added as solid) for 2 hours at 4°C on a tube roller. The precipitate was then centrifuged at 25,000 rpm for 20 min at 4°C (Beckman Coulter Avanti J-25), and pellets were resuspended in HB100 buffer equal to the initial volume of clarified cell lysate. This resuspension was then incubated with Ni^{2+}-charged Fractogel (Merck) overnight at 4°C on a tube roller. On the following day, beads with bound proteins were washed sequentially with at least 40 column volumes of HB100 buffer and eluted with 300 mM imidazole in HB100. Protein fractions were then concentrated by centrifugal filtration as above and loaded onto a Superose 6 10/300 column (GE Healthcare) with HB100 as column buffer. Fractions were analyzed by SDS-PAGE, and protein purity confirmed by Coomassie Blue staining.

Assays for protein copurification with GST-Mto1[bonsai] were performed by first coinfecting High Five cells with equal volumes of V_2 virus for GST-Mto1[bonsai], Mto2, and γ-TuSC (untagged), with and without V_2 virus for Mzt1. Cell pellets were lysed as above, and clarified cell lysates were incubated with glutathione agarose (Sigma-Aldrich) overnight at 4°C on a tube roller. The next day, agarose beads with bound proteins were washed with a minimum of 40 column volumes of HB100 buffer, and proteins were eluted from the beads by heating at 95°C in Laemmli sample buffer.

Assays for protein copurification with Strep-Mto1/2[bonsai] were performed similar to GST-Mto1[bonsai] coexpression, by coinfecting High Five cells with equal volumes of V_2 virus for Strep-Mto1/2[bonsai] and γ-TuSC (untagged), with and without V_2 virus for Mzt1. Clarified cell lysates were incubated with Strep-Tactin Sepharose beads (IBA) overnight at 4°C on a tube roller. Bound proteins were eluted from the beads by heating at 95°C in Laemmli sample buffer and analyzed by SDS-PAGE and western blot to assay proteins that copurified. In parallel experiments, proteins were released from the beads either by cleavage of the tag by rhinovirus 3C protease or by elution with biotin in HB100 buffer.

To assay copurification of proteins with Mto1[bonsai], eluted samples were analyzed qualitatively by SDS-PAGE and Coomassie Blue or SYPRO Ruby staining, and quantitatively by western blotting. Western blots were probed with homemade sheep anti-Mto1 (1:1000) [14], anti Mto2 (1:1000) [45], anti-Alp6 (1:1000) [16], anti-Alp4 (1:1000) [16], and anti-Mzt1 (1:1000; this study) antisera, followed by unlabelled GT-34 mouse monoclonal anti-goat antibody (1:10000) (Sigma) and IRDye800CW donkey anti-mouse antibody (1:5000). γ-tubulin was detected using monoclonal anti-γ-tubulin antibody GTU-88 (1:5000) (Sigma) followed by anti-mouse antibody (1:5000). Blots were imaged using an Odyssey fluorescence imager (LI-COR) and quantified using Image Studio Lite (LI-COR).

In all assays for copurification of untagged proteins with tagged proteins (i.e., after coexpression by coinfection), untagged proteins were always expressed in excess, based on western blotting of unbound proteins after incubation of clarified cell lysates with glutathione- or Strep-Tactin-agarose beads. Therefore, slight variations in "input" levels between different coinfection samples (e.g., Figure S4C) are not expected to affect results.

Except where explicitly indicated (see below), purification tags on all proteins (MBP or 6xHis tags) were not removed prior to use in functional or analytical assays. The fact that MGM holocomplex is a potent nucleator *in vitro* suggests that neither the MBP tag on Alp6 nor the 6xHis tags on Mto1[bonsai] or Mzt1 negatively affects their function. In this context, we also note that in assays for copurification of γ-TuSC proteins in the absence versus presence of Mzt1 (Figures 4 and S4), Alp6 was untagged.

For experiments in which tags were removed, MGM complexes were treated with 6xHis-tagged tobacco etch virus (TEV) protease, to cleave the MBP-6xHis tag from the C terminus of the Alp6-MBP protein and the 6xHis-tags from the N- and C- terminus of Mto1[bonsai] and Mzt1 proteins, respectively. TEV was added to MGM complex at a 1:85 (TEV:MGM) ratio (w/w) and incubated at 4°C for 16 hours to ensure optimal cleavage of the tags. Removal of the cleaved MBP-His and His-tags, as well as (uncleaved) 6xHis TEV, was achieved by binding to Ni^{2+}-charged Fractogel EMD chelate (Merck) for 3 hours at 4°C. SDS-PAGE and Coomassie staining were used to confirm purity of untagged MGM complex (which had significantly decreased yield relative to tagged MGM). The untagged MGM complex was then used for microtubule nucleation assays in the same way as tagged MGM complex (described below).

Density-gradient centrifugation

Glycerol density-gradient centrifugation was performed on affinity-purified proteins and complexes (described above). Isokinetic glycerol gradients (2 mL, 10%–30% glycerol [w/v]) were prepared in HB100 buffer using a Gradient Master 107ip (Biocomp Instruments) [23]. Gradients were loaded with 100 μL of proteins at 1 mg/mL. For analysis of the interaction between Mto1/2[bonsai] and γ -TuSC:Mzt1, 50 μL of each complex at 1 mg/mL was mixed and left to incubate at 4°C for 15 min, and the total (100 μL) was loaded onto the gradient. Gradients were centrifuged in a Beckman TLS-55 swinging bucket rotor at 55,000 rpm at 4°C. Marker proteins with known sedimentation values were analyzed in parallel, on separate gradients. 100 μL fractions were collected with a cut-off pipette tip and analyzed by SDS-PAGE and SYPRO Ruby staining (Lonza). Glycerol density-gradient sedimentation for each sample was performed twice: once at 45 min, and once at 80 min, with consistent results. A 2.5-hour centrifugation was also performed, but the sedimentation peak was too close to the pellet fraction to be able to accurately determine S-values.

Quantification of SYPRO Ruby staining was performed using a ChemiDoc Imager and Image Lab software (Bio-Rad).

Size-exclusion chromatography and SEC-MALS

Size-exclusion chromatography analysis of γ-TuSC$^{Alp6\text{-}MBP}$, γ-TuSC$^{Alp6\text{-}MBP}$:Mzt1, Alp6-MBP and Alp-6MBP:Mzt1 was performed on an ÄKTA chromatography system (GE Healthcare) using a Superose 6 10/300 column with HB100 as column buffer. 500 μL of protein was loaded on the column, with a flow rate of 0.5 mL/min, and absorbance at 280nm was recorded for one column volume. Fractions were collected in 250 μL volumes and analyzed by SDS-PAGE and Coomassie Blue staining.

To determine the molecular mass of Mzt1 in solution by size-exclusion chromatography with multi-angle light scattering (SEC-MALS), an ÄKTAmicro chromatography system (GE Healthcare) coupled to UV, static light scattering and refractive index detection (Viscotek SEC-MALS 20 and Viscotek RI Detector:VE3580; Malvern Instruments) was used. 100 μL of 1.98 mg/mL Mzt1 was run on a Superdex-75 10/300 GL size-exclusion column (GE Healthcare) pre-equilibrated in 100 mM Tris, 50mM NaCl, pH7.5 at 22°C with a flow rate of 0.5 mL/min. Light scattering, refractive index (RI) and A(280nm) were analyzed by a homo-polymer model (OmniSEC software, v 5.1; Malvern Instruments) using the following parameters for Mzt1: $\partial A / \partial c$ at 280nm 0.34 AU.mL/mg and $\partial n / \partial c$ of 0.185 mL/g.

In vitro microtubule nucleation assay

For bulk fluorescence-based microtubule polymerization assays, we used the fluorescence reporter 4',6-diamidino-2-phenylindole (DAPI) (Thermo Fisher Scientific) [30] and porcine brain tubulin (Cytoskeleton, Inc.). Tubulin stock was made at 10 mg/mL in GB (80 mM K-PIPES, pH 6.9, 2 mM MgCl2, 0.5 mM EGTA, 1 mM GTP), and DAPI stock made at 10 μM DAPI in GB. Reactions were set up on ice by mixing purified recombinant protein complexes (in a total volume of 5 μL) with 10 μL of tubulin stock and 35 μL DAPI stock (final volume, 50 μL). Final assay composition was 80 mM K-PIPES, pH 6.9, 2 mM MgCl2, 0.5 mM EGTA, 1 mM GTP, 7 μM DAPI, 2 mg/mL porcine tubulin. Reactions were then transferred into a 96-well half area black microplate (Greiner Bio-One, Inc.) and measured on a SpectraMax M5 multi-mode microplate reader (Molecular Devices) at 360 nm excitation and 450 nm emission at 37°C. Readings were acquired every minute for two hours.

For fluorescence microscopy-based microtubule polymerization assays, we used a 1:10 mix of rhodamine-labeled porcine brain tubulin (Cytoskeleton, Inc.) to unlabeled porcine brain tubulin. Mixed tubulin stock was made at a total concentration of 5 mg/mL in GB. Reactions were set up by mixing 1 μL of purified recombinant protein complexes, 4 μL of mixed tubulin stock (final concentration 2 mg/mL) and 5 μL of GB, to a final reaction volume of 10 μL, and incubated at 37°C. 1 μL aliquots were taken at 10-min intervals and diluted 1:10 in prewarmed fixing solution (50% glycerol plus 0.1% glutaraldehyde, in GB without GTP). 2.5 μL of this reaction was then spotted onto a glass slide, covered with a 24x50 mm coverslip (VWR), and randomly-selected fields were imaged with a 63x oil-immersion objective on an LSM880 laser scanning microscope with Airyscan (Zeiss).

For both the DAPI microtubule polymerization assays (e.g., Figure 3A), the molar concentration of MGM holocomplex was estimated based on the assumption that the MGM holocomplex forms a γ-TuRC-like ring complex consisting of 6.5 γ-TuSCs (accounting for 13 γ-tubulins per turn, with a half γ-TuSC overlap) [18]. Our previous work also indicated that within each actively-nucleating punctum *in vivo*, there are ~2 copies of Mto1[bonsai] and ~2 copies of Mto2 per γ-TuSC [23], consistent with other work in budding yeast [31]. Therefore, our estimate for the molar concentration of the MGM complex (derived from μg/mL) assumes 6.5 copies of Alp6-MBP, 6.5 copies of Alp4, 13 copies of Gtb1, 13 copies of Mto1/2[bonsai] and 6.5-13 copies of Mzt1 (because Mzt1 is a very small protein, its mass does not contribute significantly to the total mass of MGM).

Microscopy image analysis

ImageJ (Fiji; NIH) was used to process all raw microscopy images. Images shown are maximum projections of 10 Z sections with 0.2 μm step-size. To quantify microtubule polymerization, the total fluorescence signals from all microtubules within an individual field were summed and plotted for each time point, using GraphPad Prism.

Antibody production

Antibodies against *S. pombe* Mzt1 were generated using GST-Mzt1-6xHis and Mzt1-6xHis fusion proteins expressed in *E. coli*. The Mzt1 coding sequence was codon-optimized for *E. coli* expression and synthesized by GeneArt, as plasmid pKS1809. Expression plasmids were generated by Gateway cloning (Thermo Fisher Scientific), using vectors p0GWA (for Mzt1-6xHis) or pGGWA (for GST-Mzt1-6xHis), kindly provided by Didier Busso [40]. First, PCR was used to amplify the Mzt1 coding sequence with a C-terminal rhinovirus 3C protease cleavage site and flanking attB1/2 sites (for Mzt1-6xHis; using primers OKS2876 and OKS2875) or with an additional N-terminal TEV protease cleavage site (for GST-Mzt-6xHis; using primers OKS2874 and OKS2875). PCR products were integrated into the Gateway donor vector pDONR201 using BP clonase (Thermo Fisher Scientific), and then transferred, using LR clonase (Thermo Fisher Scientific), into p0GWA to generate the Mzt1-6xHis expression plasmid pKS1821, or into pGGWA to generate the GST-Mzt1-6xHis expression plasmid pKS1822. Recombinant proteins were expressed in BL21-CodonPlus (DE3)-RIL *E.coli* (Agilent) by induction with 1mM IPTG for 18 hours at 18°C.

Recombinant proteins were purified from inclusion bodies via the 6xHis-tag, under denaturing conditions. Proteins were further purified by SDS-PAGE, and the gel slice was lyophilized and ground to a fine powder prior to immunization. Antibodies raised against GST-Mzt1-6xHis gave the best signal on western blots; crude anti-Mzt1 sheep serum produced a single band of the expected size on western blots, without the need for further affinity purification (e.g. Figure S2B), and no band was observed in negative controls.

Crosslinking mass spectrometry

For cross-linking mass spectrometry, MGM holocomplex and Alp6-MBP:Mzt1 complex were cross-linked (independently) with either 1-ethyl-3-(3-dimethylaminopropyl)carbodiimide hydrochloride (EDC)/N-hydroxysulfosuccinimide (Sulfo-NHS) or sulfosuccinimidyl 4,4'-azipentanoate (Sulfo-SDA) (Thermo Fisher Scientific). The MGM complex was incubated with EDC and Sulfo-NHS at a 1:2:4.4 (w/w/w) ratio for 90 min at 18°C in 40 mM K-HEPES, 300 mM NaCl, 1 mM $MgCl_2$ pH7.5. Alp6-MBP:Mzt1 complex was incubated with either EDC/Sulfo-NHS at a 1:6:13.2 (w/w/w) ratio for 90 min at 18°C, or with Sulfo-SDA at a 1:0.3 (w/w) ratio for 1 hour on ice with a further 30 min of UV irradiation. The mixtures were separated on a 4%–12% Bis-Tris polyacrylamide precast gel

(Bolt; Thermo Fisher Scientific), stained with Coomassie Blue at room temperature for 1 hour, and destained with 10% acetic acid overnight. On the following day, gels were washed twice in MilliQ water for 30 min each. The higher molecular-weight bands (> 250 kDa; for both complexes) and Alp6-MBP bands (for experiments involving Alp6-MBP:Mzt1) were excised with a clean scalpel for further analysis.

Excised gel bands were first destained by incubating with 50 mM ammonium bicarbonate (Sigma-Aldrich) and 100% acetonitrile at a 1:1 ratio (v/v) for 1 hour at 37°C with shaking. Proteins were then digested with trypsin, following previously established protocols [46]. Briefly, proteins were first reduced with 10 mM DTT (Sigma-Aldrich) for 30 min at 37°C in a shaker and alkylated with 55 mM iodoacetamide (Sigma-Aldrich) for 20 min at room temperature, followed by trypsin digestion overnight at 37°C (Thermo Fisher Scientific).

Peptides from the MGM holocomplex were fractionated after digestion using SCX-Stage-Tips [47, 48]. In brief, peptide mixtures were loaded on a SCX-Stage-Tip with loading buffer (0.5% v/v acetic acid, 20% v/v acetonitrile, 50 mM ammonium acetate). The bound peptides were eluted into two fractions, with buffers containing 100 mM ammonium acetate and 500 mM ammonium acetate, respectively. These peptide fractions were subsequently desalted using C18-StageTips [49] for LC-MS/MS analysis.

Peptides from the Alp6-MBP:Mzt1 complex were loaded onto StageTips for desalting and eluted in 80% acetonitrile in 0.1% TFA prior to LC-MS/MS analysis.

For the MGM holocomplex, LC-MS/MS analysis was performed using Orbitrap Fusion Lumos (Thermo Fisher Scientific) with a "high/high" acquisition strategy. Peptide separation was carried out on an EASY-Spray column (50 cm × 75 μm i.d., PepMap C18, 2 μm particles, 100 Å pore size, Thermo Fisher Scientific). Mobile phase A consisted of water and 0.1% v/v formic acid. Mobile phase B consisted of 80% v/v acetonitrile and 0.1% v/v formic acid. Peptides were loaded at a flow rate of 0.3 μL/min and eluted at 0.2 μL/min using a linear gradient from 2% mobile phase B to 40% mobile phase B over a period of 109 min (for MGM holocomplex fractions) or 139 min (for Alp6-MBP:Mzt1), followed by a linear increase from 40% to 95% mobile phase B over a period of 11 min. The eluted peptides were directly introduced into the mass spectrometer.

MS data were acquired in the data-dependent mode with 3 s acquisition cycle. Precursor spectrum was recorded in the Orbitrap with a resolution of 120,000. The ions with a precursor charge state between 3+ and 8+ were isolated with a window size of 1.6 m/z and fragmented using high-energy collision dissociation (HCD) with collision energy 30. The fragmentation spectra were recorded in the Orbitrap with a resolution of 15,000. Dynamic exclusion was enabled with single repeat count and 60 s exclusion duration.

The mass spectrometric raw files were processed into peak lists using MaxQuant (version 1.5.3.30) [50], and cross-linked peptides were matched to spectra using Xi software (version 1.6.745) (https://github.com/Rappsilber-Laboratory/XiSearch). Protein sequences for searches were obtained from the *S. pombe* database (PomBase; August 2013 release; https://www.pombase.org) [51], with added sequences for relevant tags (e.g., MBP tag on Alp6). Search parameters were: MS accuracy, 3 ppm; MS/MS accuracy, 10ppm; enzyme, trypsin; cross-linker, EDC; max missed cleavages, 4; missing mono-isotopic peaks, 2; fixed modification, carbamidomethylation on cysteine; variable modifications, oxidation on methionine and phosphorylation on serine; fragments, b and y ions with loss of H_2O, NH_3 and CH_3SOH. FDR was estimated using XiFDR on 5% residue level [52].

Fission yeast strain construction

A fission yeast strain was constructed to express FLAG-tagged Mzt1 at the endogenous *mzt1* locus under the control of the thiamine-repressible *nmt81* promoter [53], because previous work showed that repression of *nmt81*-driven *mzt1* compromises *mzt1* function *in vivo* [11]. We generated an *nmt81*:3xFLAG-Mzt1 strain by PCR-based gene tagging, as follows. The plasmid pFA6a-hphMX6-P81nmt1-3FLAG (pKS1284; Addgene #19350 [41] was purchased from Addgene and amplified by PCR using oligonucleotides OKS2671 (forward primer) and OKS2672 (reverse primer) and Phusion DNA polymerase (New England Biolabs). Purified PCR product was used to transform strain KS516, followed by selection for stable-integrant hygromycin-resistant strains that showed normal growth on EMMG (also known as PMG) minimal medium plates [43] lacking thiamine and poor or no growth on equivalent plates containing 15 μM thiamine (Figure S4I). The resulting strain was named KS7623. Repression of 3xFLAG-Mzt1 expression in the presence of thiamine was confirmed by anti-FLAG western blotting (Figure S4J). Because our anti-Mzt1 antibody used to detect recombinant protein was not sufficiently sensitive to detect untagged Mzt1 in fission yeast, the expression level of non-repressed *nmt81*:3xFLAG-Mzt1 relative to untagged Mzt1 (under control of the endogenous *mzt1*+ promoter) is not known. The *mto1[bonsai]-mECitrine* strain was also constructed by PCR-based gene tagging, using template plasmid pFA6a-mEcitrine-KanMX6 (pKS1330; Addgene #105150) [42] and oligonucleotides OKS2105 (forward primer) and OKS2116 (reverse primer). Purified PCR product was used to transform strain KS516. The resulting strain, KS7462, which expresses Mto1[bonsai]-mECitrine under control of the endogenous *mto1* promoter at the *mto1* locus, was confirmed by colony PCR, microscopy and western blotting. Strains used for co-immunoprecipitation experiments (i.e., strains KS10055 and KS10059) were derived from KS7623 and KS7462 by conventional genetic crosses [54].

Fission yeast coimmunoprecipitation

Mto1[bonsai] immunoprecipitation (IP) experiments from *nmt81*:3xFLAG-Mzt1 and control strains were performed using strains KS10059 and KS10055, respectively. In text and figures, we refer to the control strain KS10055 as "wild-type." Both strains were grown in minimal EMMG medium with supplements to mid-log phase, harvested, washed with STOP buffer (10 mM EDTA, 50 mM NaF, 150 mM NaCl, 1 mM NaN_3), resuspended in water and snap-frozen in liquid nitrogen. For *mzt1* shut-off and control experiment, 20 μM thiamine were added to the PMG medium for 16 hr. Cryogrinding of frozen cells was performed under liquid nitrogen in a 6870 Freezer/Mill® (SPEX SamplePrep), using sample pre-cool for 2 min, 10 rounds of "run" and "cool" cycle each

for 2 min, with beat rate = 10. 3.74 g of frozen cell powder was thawed for 20 min at 4°C in 7.5 mL IP buffer (50 mM Tris-HCl pH 7.5, 50 mM NaF, 150 mM NaCl, 20 mM Sodium beta-glycerophosphate, 0.2% Triton X-100, 1 mM sodium orthovanadate, 1 mM EDTA, 10 μg/mL of each 'CLAAPE' protease inhibitors, 2 mM AEBSF, 2 mM PMSF). Cell lysate was then clarified by two cycles of centrifugation (4500 rpm; 15 min each cycle) at 4°C, and the pellets were discarded. 164 μL of clarified cell lysate was mixed with equal volume of Laemmli sample buffer (containing 1% beta-mercaptoethanol), heated to 95°C for 3 min and kept as an "input" sample. The remainder was mixed with 150 μL Protein G Dynabeads slurry (Thermo Fisher Scientific; 10003D) that had been previously cross-linked to homemade affinity-purified sheep anti-GFP antibody using dimethylpimelimidate [55], and the mixture incubated for 1 hr at 4°C with gentle rotation, followed by washing three times with 500 μL IP buffer. Dynabeads were then incubated with Laemmli buffer (without reducing agent), and heated to 95°C for 3 min. Eluates from Dynabeads were then supplemented with beta-mercaptoethanol (0.5% v/v final). For SDS-PAGE, gel lanes were loaded with either 10 μL of input samples (corresponding to 5 μL of original clarified lysate) or 10 μL of IP eluates (corresponding to 535 μL of original clarified lysate), except for Alp4 and Alp6, which used double the amounts of each. Proteins were transferred to nitrocellulose by wet transfer. Western blots were stained with Ponceau S and scanned prior to blocking in 5% milk in TBS-T (TBS buffer containing 0.1% TWEEN® 20). Western blots were probed with homemade sheep anti-Mto1 and anti-Mto2 antisera (both at 1:1,000), affinity-purified homemade sheep anti-Alp4 and anti-Alp6 antibodies (both at 1:100), mouse monoclonal anti-γ-tubulin antibody (clone GTU-88, Sigma, T6557; at 1:5,000) or mouse monoclonal anti-FLAG antibody (clone M2, Sigma, F1804) in TBS-T plus 2% non-fat milk. Blots involving sheep primary antibodies were washed and then incubated with mouse monoclonal anti-goat/sheep IgG antibody (clone GT-34, Sigma, G2904, at 1:10,000) to allow recognition by labeled anti-mouse antibody. All blots were then washed and incubated with IRDye 800CW Donkey anti-Mouse IgG (LI-COR, 926-32212, at 1:5,000) in TBS-T plus 2% non-fat milk. Blots were imaged using an Odyssey CLx fluorescence imager (LI-COR) and quantified using Image Studio Lite (LI-COR).

Electron microscopy

For negative stain EM, 4 μl of MGM holocomplex (including MBP tag on Alp6) at 170 μg/mL was added to freshly glow-discharged copper grids (400 mesh, continuous carbon film; TAAB) and incubated for two minutes. Grids were blotted with filter paper (Grade 1; Whatman) and washed twice by touching with 15 μL buffer (40 mM KHEPES, pH 7.5, 100 mM KCl) and blotting after each wash. Grids were then stained by touching a 15 μL droplet of 2% uranyl acetate solution two times, each time followed by immediate blotting, and then touching a third time, followed by incubation for 2 min. Excess stain was then blotted away carefully, and grids were air-dried and stored until imaged.

Electron microscopy was performed on a JEM-1400 transmission electron microscope (JEOL) operating at 80 kV, equipped with a OneView 4k x 4k CMOS detector (Gatan Inc.) Images were recorded with a pixel size of 2.15 Å, and a defocus of −1 μm. images were processed using ImageJ software.

Figure preparation

Images for figures were prepared using ImageJ (NIH), Photoshop (Adobe), Excel (Microsoft) and GraphPad Prism (GraphPad). Figures were compiled using Illustrator (Adobe)

QUANTIFICATION AND STATISTICAL ANALYSIS

Quantification of tubulin polymerization by DAPI fluorescence

For tagged complexes, DAPI tubulin polymerization with: (i) 4.5 μg/mL MGM complex was performed 7 times; (ii) 0.45 μg/mL MGM complex was performed once; (iii) 3.2 μg/mL γ-TuSC:Mzt1 complex was performed 6 times; (iv) 0.32 μg/mL γ-TuSC:Mzt1 was performed once; (v) tubulin alone was performed 7 times; (vi) 3.8 μg/mL γ-TuSC was performed once; (vii) 4.35 μg/mL γ-TuSC:Mto1/2[bonsai] was performed once; (viii) 0.86 μg/mL Mto1/2[bonsai] was performed twice; and (ix) 2 μM paclitaxel was performed twice. To compare nucleation activity of tagged complexes with untagged complexes (which had much lower yield), DAPI tubulin polymerization with: (i) 1.4 μg/mL tagged MGM complex; (ii) 1.4 μg/mL untagged MGM complex; and (iii) 1.7 μg/mL untagged γ-TuSC:Mto1/2[bonsai] was performed once.

Quantification of microtubules visualized by fluorescence microscopy

Total combined fluorescence signal for all microtubules within an image field was quantified for each random image taken, using ImageJ software, and a total of ten measurements were plotted for each time point and condition, using GraphPad Prism software (GraphPad). For experiments involving tagged complexes, microtubule polymerization for the MGM holocomplex with tubulin and γ-TuSC$^{Alp6\text{-}MBP}$:Mzt1 complex with tubulin were performed four times, "tubulin alone" was performed twice, and Mto1/2[bonsai complex] with tubulin was performed once. For experiments involving untagged complexes, all experiments were performed once. Counts of microtubule numbers were performed manually, using ten fields for tagged complexes and seven fields for untagged complexes.

Quantification of copurification by western blot and Coomassie Blue staining

Assays for copurification of Mto2 and γ-TuSC with GST-Mto1[bonsai] and Strep-Mto1[bonsai] in the presence and absence of Mzt1 (Figures 4D and S4D) were quantified from western blots using Image Studio Lite software (LI-COR). Copurification assay with GST-Mto1[bonsai] was performed once for all proteins analyzed. For copurification assays with Strep-Mto1[bonsai], quantification of Alp6,

Alp4 and Mto1[bonsai] was performed 4 times, quantification of Gtb1 was performed three times, and quantification of Mto2 was performed once (because in some experiments the Mto2 signal was too high, out of linear range). Error bars show SEM. Coimmunoprecipitation experiments from fission yeast cell lysates were performed once.

Levels of Mto1[bonsai] relative to Alp4 or Alp6 (Figure 4B) after purification of the γ-TuSC$^{Alp6\text{-}MBP}$:Mto1/2[bonsai] complex in the presence and absence of Mzt1 were quantified from Coomassie Blue staining, using Image Studio Lite software (LI-COR).

DATA AND SOFTWARE AVAILABILITY

The mass spectrometry proteomics data have been deposited to the ProteomeXchange Consortium via the PRIDE [56] partner repository (https://www.ebi.ac.uk/pride/archive) with the dataset identifier ProteomeXchange: PXD012624.

Current Biology, Volume *29*

Supplemental Information

Reconstitution of Microtubule Nucleation *In Vitro* Reveals Novel Roles for Mzt1

Su Ling Leong, Eric M. Lynch, Juan Zou, Ye Dee Tay, Weronika E. Borek, Maarten W. Tuijtel, Juri Rappsilber, and Kenneth E. Sawin

A

	S. pombe	*S. cerevisiae*	*C. albicans*	*H. sapiens*	*D. melanogaster*
γ-TuSC proteins	Gtb1	Tub4	*Ca*Tub4	γ-tubulin	γ-tubulin
	Alp4	Spc97	*Ca*Spc97	GCP2	Grip84
	Alp6	Spc98	*Ca*Spc98	GCP3	Grip91
γ-TuRC-specific proteins	Gfh1	(none)	(none)	GCP4	Grip75
	Mod21	(none)	(none)	GCP5	Grip128
	Alp16	(none)	(none)	GCP6	Grip163
Mzt1	Mzt1	(none)	*Ca*Mzt1	MOZART1	Mzt1
CM1 proteins	Pcp1<br>Mto1	Spc110<br>Spc72	*Ca*Spc110<br>*Ca*Spc72	CDK5RAP2<br>myomegalin	Cnn
(Mto1 interactor & possible multimerizer)	(Mto2)	?	?	?	?

B

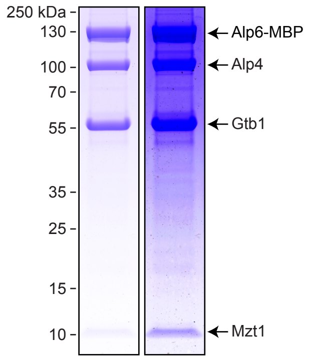

C

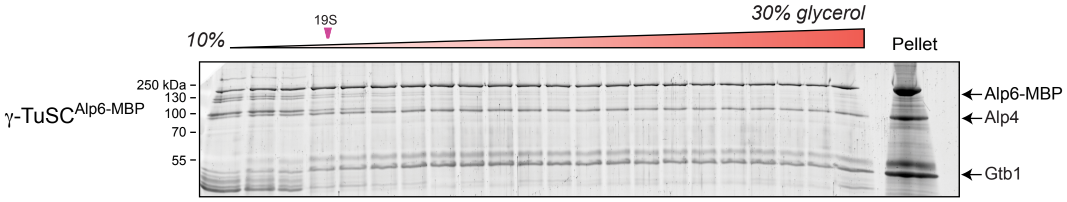

D

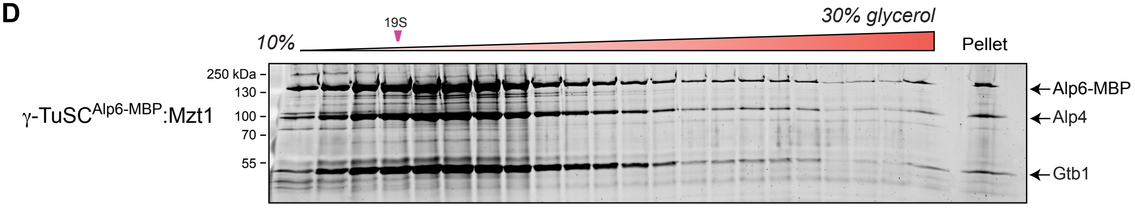

(Mzt1 not imaged)

Figure S1. Further characterization of γ-TuSC$^{Alp6-MBP}$ and γ-TuSC$^{Alp6-MBP}$:Mzt1. Related to Figure 1.

(A) Nomenclature for the conserved γ-tubulin complex proteins and regulators relevant to this study. **(B)** SDS-PAGE of γ-TuSC$^{Alp6-MBP}$:Mzt1, shown at the two different contrast levels used for the composite in Figure 1D. **(C)** SDS-PAGE of 45-minute density-gradient centrifugation of γ-TuSC$^{Alp6-MBP}$ (SYPRO Ruby stain). Pelleting of γ-TuSC$^{Alp6-MBP}$ under these conditions indicates a sedimentation coefficient of at least 150S. See also Figure 1C. **(D)** SDS-PAGE of 45-minute density-gradient centrifugation of γ-TuSC$^{Alp6-MBP}$:Mzt1 (SYPRO Ruby stain). Because of the amount of protein loaded, Mzt1 was not visualized in this experiment. See also Figure 1F.

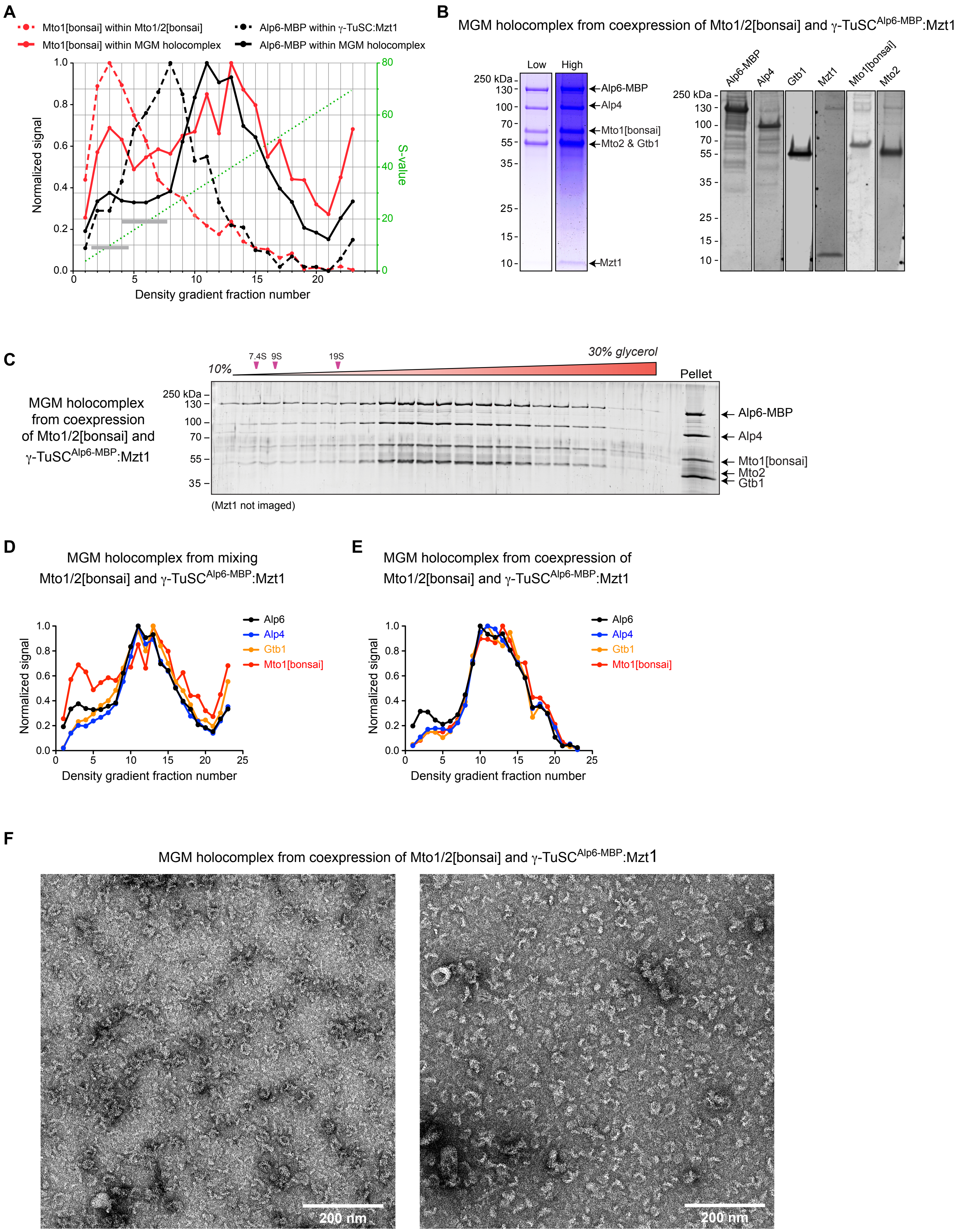
A
Mto1[bonsai] within Mto1/2[bonsai]
Mto1[bonsai] within MGM holocomplex
Alp6-MBP within γ-TuSC:Mzt1
Alp6-MBP within MGM holocomplex
Normalized signal
S-value
Density gradient fraction number
B
MGM holocomplex from coexpression of Mto1/2[bonsai] and γ-TuSC^Alp6-MBP:Mzt1
Low
High
250 kDa
Alp6-MBP
Alp4
Mto1[bonsai]
Mto2 & Gtb1
Mzt1
Gtb1
Mto2
C
10%
30% glycerol
7.4S
9S
19S
Pellet
MGM holocomplex from coexpression of Mto1/2[bonsai] and γ-TuSC^Alp6-MBP:Mzt1
(Mzt1 not imaged)
D
MGM holocomplex from mixing Mto1/2[bonsai] and γ-TuSC^Alp6-MBP:Mzt1
Alp6
Alp4
Gtb1
Mto1[bonsai]
E
MGM holocomplex from coexpression of Mto1/2[bonsai] and γ-TuSC^Alp6-MBP:Mzt1
F
MGM holocomplex from coexpression of Mto1/2[bonsai] and γ-TuSC^Alp6-MBP:Mzt1
200 nm
200 nm

Figure S2. Further characterization of MGM holocomplex. Related to Figure 2.
(A) Comparison of density-gradient sedimentation profiles of Mto1[bonsai] when present in Mto1/2[bonsai] complex (dashed red line; quantification of Figure 2A) vs. when present in MGM holocomplex generated by mixing Mto1/2[bonsai] with γ-TuSC$^{Alp6\text{-}MBP}$:Mzt1 (solid red line; quantification of Figure 2B), and of Alp6-MBP when present in γ-TuSC$^{Alp6\text{-}MBP}$:Mzt1 (dashed black line; quantification of Figure 1F) vs. when present in MGM holocomplex generated by mixing (solid black line; quantification of Figure 2B). Signals for a given protein were normalized to the highest signal for that protein in the gradient. Upon mixing to generate the MGM holocomplex, Mto1[bonsai] and Alp6-MBP (representative of Mto1/2[bonsai] and γ-TuSC$^{Alp6\text{-}MBP}$:Mzt1 respectively) cosediment, with significant sedimentation shifts compared to when they are in separate complexes. Gray bars indicate range of full-width half-maximal sedimentation of protein standards β-amylase (9S) and thyroglobulin (19S). Dashed green line shows relationship between fraction number and estimated S-value (right-hand Y-axis), based on isokinetic sedimentation. **(B)** SDS-PAGE (Coomassie Blue stain) and western blotting of MGM holocomplex purified from coexpression of Mto1/2[bonsai] with γ-TuSC$^{Alp6\text{-}MBP}$:Mzt1 (amylose purification). Gel stain is shown at two different contrast levels, to visualize both high and low molecular-weight proteins. Western blotting confirms identity of different proteins. Note comigration of Mto2 and γ-tubulin (Gtb1) on this gel system. **(C)** SDS-PAGE of 80-minute density-gradient centrifugation of MGM purified from coexpression of Mto1/2[bonsai] and γ-TuSC$^{Alp6\text{-}MBP}$:Mzt1 (SYPRO Ruby stain). Sedimentation of MGM purified from coexpression is essentially identical to that of MGM generated by combining independent complexes (Figure 2B). Because of the amount of protein loaded, Mzt1 was not visualized in this experiment. **(D, E)** Density-gradient sedimentation profiles of proteins in MGM generated by mixing Mto1/2[bonsai] and γ-TuSC$^{Alp6\text{-}MBP}$:Mzt1 (D) and in MGM purified from coexpression of Mto1/2[bonsai] and γ-TuSC$^{Alp6\text{-}MBP}$:Mzt1 (E). Data are from quantification of SYPRO Ruby staining shown in Figures 2B and S2C. Signals were normalized as in A. Very similar profiles would suggest that relative stoichiometry of MGM proteins does not change through the gradient. In D, the relative stoichiometry of MGM proteins is mostly constant throughout the peak. An excess of Mto1[bonsai] in the first few fractions may be the result of mixing γ-TuSC$^{Alp6\text{-}MBP}$:Mzt1 with a slight excess of Mto1/2[bonsai] (which itself peaks in fraction 3, as shown in A). A small excess of Alp6 in the first few fractions is likely due to fact that the MBP tag on Alp6 was used for purification of γ-TuSC$^{Alp6\text{-}MBP}$:Mzt1; as a result, purified γ-TuSC$^{Alp6\text{-}MBP}$:Mzt1 may have a small amount of Alp6 that is not in complex with other γ-TuSC proteins. In E, the relative stoichiometry of MGM proteins is essentially constant across the gradient. A small excess of Alp6 in the first few fractions is likely due to the reason mentioned above. In this analysis, Mto2 was not quantified because its multiple bands (due to multiple phosphorylation states; [S1]) are less distinct and are close to Gtb1**.** **(F)** Negative-stain electron microscopy of MGM purified from coexpression of Mto1/2[bonsai] and γ-TuSC$^{Alp6\text{-}MBP}$:Mzt1. Complete uncropped image fields are shown. Image shown in Figure 2D is taken from center of field on left. Field on right has higher contrast but worse preservation of morphology.

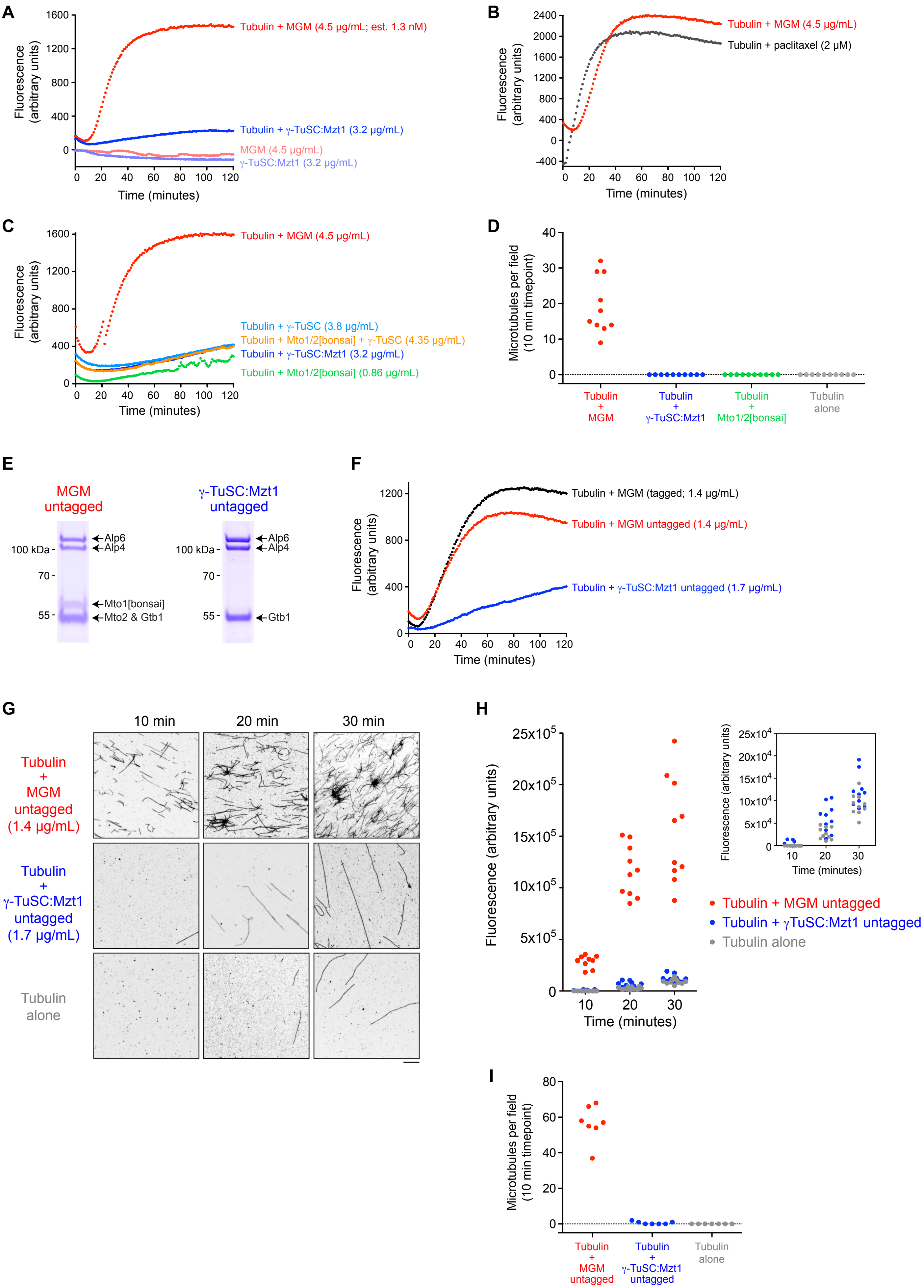

A
Fluorescence (arbitrary units)
Time (minutes)
Tubulin + MGM (4.5 μg/mL; est. 1.3 nM)
Tubulin + γ-TuSC:Mzt1 (3.2 μg/mL)
MGM (4.5 μg/mL)
γ-TuSC:Mzt1 (3.2 μg/mL)
B
Tubulin + MGM (4.5 μg/mL)
Tubulin + paclitaxel (2 μM)
C
Tubulin + MGM (4.5 μg/mL)
Tubulin + γ-TuSC (3.8 μg/mL)
Tubulin + Mto1/2[bonsai] + γ-TuSC (4.35 μg/mL)
Tubulin + γ-TuSC:Mzt1 (3.2 μg/mL)
Tubulin + Mto1/2[bonsai] (0.86 μg/mL)
D
Microtubules per field (10 min timepoint)
Tubulin + MGM
Tubulin + γ-TuSC:Mzt1
Tubulin + Mto1/2[bonsai]
Tubulin alone
E
MGM untagged
γ-TuSC:Mzt1 untagged
100 kDa
70
55
Alp6
Alp4
Mto1[bonsai]
Mto2 & Gtb1
Gtb1
F
Tubulin + MGM (tagged; 1.4 μg/mL)
Tubulin + MGM untagged (1.4 μg/mL)
Tubulin + γ-TuSC:Mzt1 untagged (1.7 μg/mL)
G
10 min
20 min
30 min
Tubulin + MGM untagged (1.4 μg/mL)
Tubulin + γ-TuSC:Mzt1 untagged (1.7 μg/mL)
Tubulin alone
H
Fluorescence (arbitrary units)
Time (minutes)
Tubulin + MGM untagged
Tubulin + γTuSC:Mzt1 untagged
Tubulin alone
I
Microtubules per field (10 min timepoint)
Tubulin + MGM untagged
Tubulin + γ-TuSC:Mzt1 untagged
Tubulin alone

Figure S3. Additional controls for assays of microtubule nucleation by MGM holocomplex. Related to Figure 3.

(A) DAPI fluorescence assay showing that in the absence of tubulin, neither MGM nor γ-TuSC$^{Alp6\text{-}MBP}$:Mzt1 causes an increase in fluorescence. This indicates that fluorescence increase is due to microtubule (MT) polymerization. Data for tubulin plus MGM and tubulin plus γ-TuSC$^{Alp6\text{-}MBP}$:Mzt1 are reproduced from Figure 3A. **(B)** DAPI fluorescence assay for MT polymerization, comparing activity of MGM holocomplex with 2 μM paclitaxel (positive control for MT assembly). **(C)** DAPI fluorescence assay showing that subcomplexes of MGM are insufficient for MT nucleation. Different "μg/mL" concentrations were used in order to have comparable molarities for the different protein complexes. Discontinuity in red trace was due to an air bubble. **(D)** Quantification of MT number (MTs per microscope field) for experiments shown in Figure 3B. Only the 10-minute timepoint is shown, because MT bundling or clumping at later timepoints precluded accurate counts in most fields. **(E)** SDS-PAGE of MGM and γ-TuSC:Mzt1 after removal of MBP-6xHis tag from Alp6-MBP and 6xHis-tags from Mto1[bonsai] and Mzt1 proteins. The resulting untagged complexes were used for experiments in (F-H). **(F)** DAPI fluorescence assay showing that untagged MGM and tagged MGM have comparable effects on MT nucleation. **(G)** Rhodamine-tubulin fluorescence microscopy assay for MT nucleation, using the indicated untagged complexes at the same concentrations as in F. **(H)** Quantification of MT polymerization for experiments in G. Each data point represents total MT fluorescence within a randomly-chosen field. Inset shows expanded scale for tubulin plus untagged γ-TuSC:Mzt1 and for tubulin alone. **(I)** Quantification of MT number (MTs per microscope field) for experiments in G. Only the 10-minute timepoint is shown. Note that experiments in F-H used a different batch of tubulin compared to those shown in A-D and in Figure 3; therefore, results from the two sets of experiments should not be compared directly. Scale bar, 10 μm.

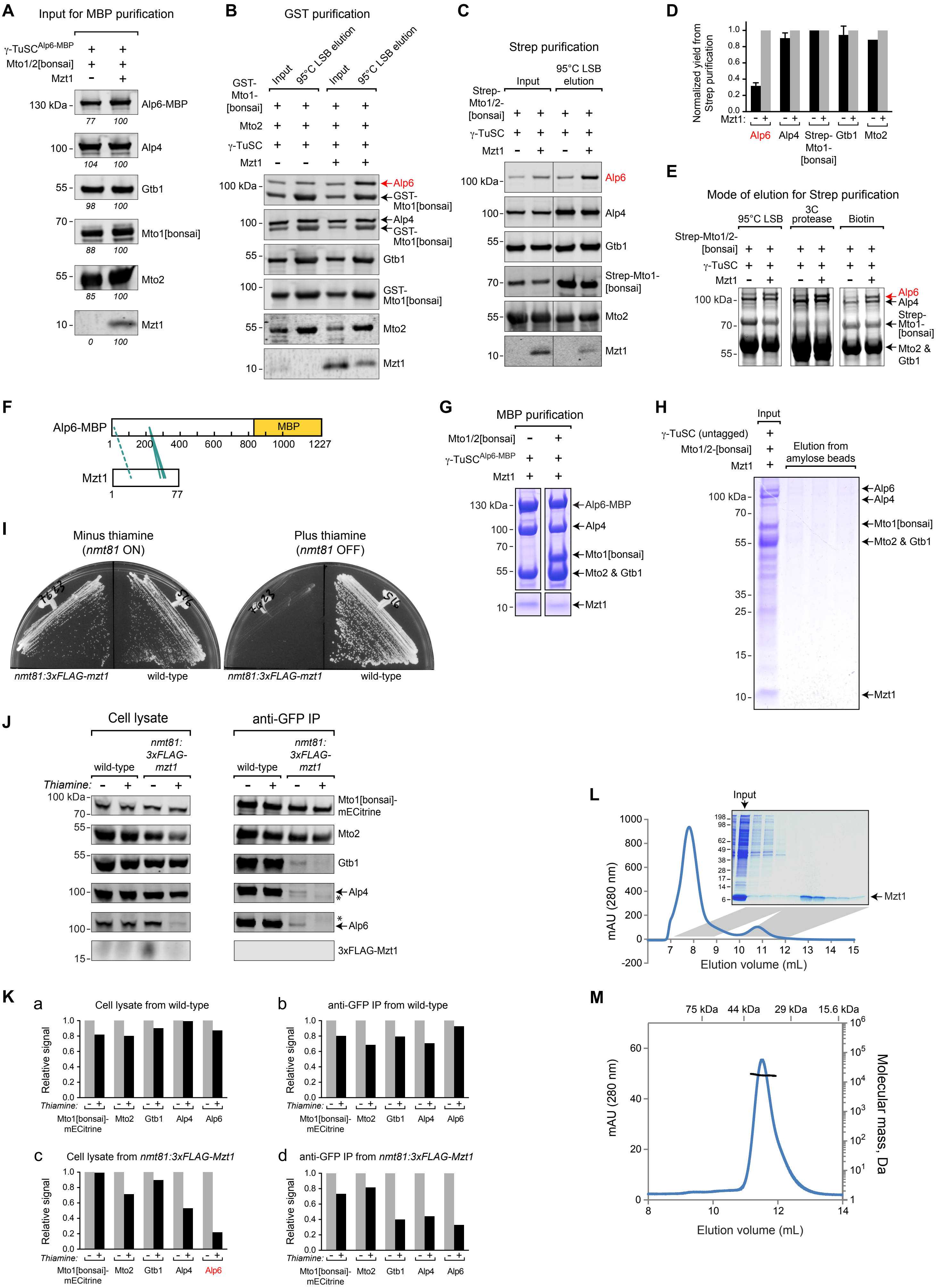
A
Input for MBP purification
B
GST purification
C
Strep purification
D
E
Mode of elution for Strep purification
F
Alp6-MBP
MBP
Mzt1
G
MBP purification
H
Input
Elution from amylose beads
I
Minus thiamine (nmt81 ON)
Plus thiamine (nmt81 OFF)
nmt81:3xFLAG-mzt1
wild-type
J
Cell lysate
anti-GFP IP
Thiamine:
K
Cell lysate from wild-type
anti-GFP IP from wild-type
Cell lysate from nmt81:3xFLAG-Mzt1
anti-GFP IP from nmt81:3xFLAG-Mzt1
Relative signal
L
mAU (280 nm)
Elution volume (mL)
M
Molecular mass, Da

Figure S4. Additional data supporting a role for Mzt1 in stabilizing Alp6 within the MGM holocomplex. Related to Figure 4.

(A) Western blots of insect cell lysates used as inputs for MBP purifications shown in Figure 4A. Numbers in italics show quantification of western blots, normalized to the signal in the presence of Mzt1 (set to 100). Mto1[bonsai] and Mto2 levels are similar in presence and absence of Mzt1. **(B)** Western blots used for quantification in Figure 4D. Because anti-Alp6 and anti-Alp4 antisera were raised against GST-fusion proteins, they also recognize GST-Mto1[bonsai]. (Affinity-purified antibodies were used only when needed to avoid excessive background bands, because affinity purification led to low yields and decreased titer.) **(C)** Western blots of insect cell lysates and elutions (95°C in Laemmli sample buffer; LSB) from Strep-Tactin-based purification of coexpressed Mto1/2[bonsai]$^{\text{Strep-Mto1[bonsai]}}$ and γ-TuSC in the absence vs. presence of Mzt1. (**D)** Quantification of western blots of the type shown in C. Error bars indicate SEM, based on multiple replicate experiments (see STAR Methods). Alp6 is not copurified as efficiently as other proteins. **(E)** SDS-PAGE of Strep-Tactin-based purification of coexpressed Mto1/2[bonsai]$^{\text{Strep-Mto1[bonsai]}}$ and γ-TuSC in the absence vs. presence of Mzt1 (SYPRO Ruby stain). In parallel experiments, proteins were eluted from Strep-Tactin beads either by heating at 95°C in LSB, by cleavage of the Strep-tag from Mto1[bonsai] using rhinovirus 3C protease, or by biotin elution. In all cases, absence of Mzt1 coexpression leads to decreased copurification of Alp6 (e.g. compared to Alp4). Additional ~50 kDa band in 3C protease elution is 3C protease itself (fused to GST). **(F)** Crosslinks between Mzt1 and Alp6-MBP identified by mass spectrometry. Solid lines indicate EDC crosslinks from Alp6 Ser219 to Mzt1 Glu62, and from Alp6 Lys220 to Mzt1 Glu56, Asp60 and Glu62. A lower-scoring EDC crosslink from Alp6 Lys307 to Mzt1 Asp60 is not shown. Dashed line indicates Sulfo-SDA crosslink from Alp6 Leu13 to Mzt1 Thr21. **(G)** SDS-PAGE of amylose purification after coexpression of the indicated proteins. Copurification of Mzt1 with γ-TuSC$^{\text{Alp6-MBP}}$ is not increased by the presence of Mto1/2[bonsai]. **(H)** SDS-PAGE showing that when Mto1/2[bonsai] and γ-TuSC:Mzt1 are coexpressed without MBP tag, no MGM proteins are purified on amylose (three right-most lanes represent successive column elutions). This is a negative control for experiment in Figure 4H. **(I)** Growth of wild-type and *nmt81:3xFLAG-mzt1* fission yeast (strains KS516 and KS7623, respectively) after two days incubation (32°C) in absence or presence of thiamine. In absence of thiamine (*nmt81* promoter derepressed), the two strains grow identically. In presence of thiamine (*nmt81* promoter repressed), *nmt81:3xFLAG*-mzt1 show negligible growth. **(J)** Western blots of clarified cell lysates and anti-GFP immunoprecipitates (IPs) from *mto1[bonsai]-mECitrine* fission yeast cells expressing either wild-type Mzt1 or *nmt81:*3xFLAG-Mzt1, in absence or presence of thiamine. Lanes for cell lysates contain 1% of the "cell-equivalents" used in lanes for anti-GFP IPs. For a given protein, the same contrast setting was used for lysates and anti-GFP IPs. Asterisks indicate non-specific bands. **(K)** Quantification of Western blots from (J). Within each graph, signals for a given protein were normalized to the signal in absence of thiamine. Three points should be noted from results in I-K. First, repression of *nmt81:3xFLAG-Mzt1* expression leads to decreased levels of Alp6 (and to some extent, Alp4) in cell lysates (graph a in K). This suggests that Mzt1 contributes to Alp6 stabilization, consistent with *in vitro* experiments. Second, repression of *nmt81:3xFLAG-Mzt1* accordingly leads to decreased coIP of γ-TuSC proteins with Mto1[bonsai]-mECitrine (graph b in K). Third, even under derepressing conditions, *nmt81:3xFLAG-Mzt1* cells show significantly decreased coIP of γ-TuSC proteins with Mto1[bonsai]-mECitrine compared to wild-type cells grown under the same conditions (IP lanes in J). This suggests that *nmt81:3xFLAG-Mzt1* is not completely functional. While this last point underscores the importance of Mzt1 for the γ-TuSC-Mto1/2[bonsai] interaction, it was also somewhat unexpected, because *nmt81:3xFLAG-Mzt1* cells grow well under depressing conditions; by contrast, *mzt1*Δ cells are inviable [S2, S3]. One explanation for this result is that *nmt81:3xFLAG-Mzt1* function, although impaired, is above the threshold required for function of γ-TuSC with Pcp1, a paralog of Mto1 that is required for intranuclear mitotic spindle formation and is essential for viability [S4, S5]. By contrast, Mto1 is required for all microtubule nucleation in the cytoplasm (and thus for normal cell morphology) but is not essential for viability [S6, S7]. **(L)** Superdex 75 size-exclusion chromatography of Mzt1 alone (without γ-TuSC). **(M)** SEC-MALS analysis of purified Mzt1. Molecular mass (19.6 kDa) indicates that Mzt1 is a dimer in solution.

**Oligonucleotide**	**Source**	**Identifier**
PCR forward primer to generate insect cell expressed Mzt-6xHis:<br>5'-GGGCTCGAGGGATCCATGTCCGAGTCCAC-3'	Eurofins	OKS2838
PCR reverse primer to generate insect cell expressed Mzt-6xHis:<br>5'-GCTAGCCTGCAGTTAGTGATGATGATGATGATGACCCTGAAAGTAAAGATTTTCGCTGGGCTC-3'	Eurofins	OKS2839
PCR forward primer to generate insect cell expressed 6xHis-Mto1[bonsai]:<br>5'-GGGGCCATGGGACATCATCATCATCATCACGAAAATCTTTACTTTCAGGGTACTACTGATGATGAACAGCTT-3'	Eurofins	OKS2182
PCR reverse primer to generate insect cell expressed 6xHis-Mto1[bonsai]:<br>5'-GGTCATGCATTTATTACTGGAGTTCTTGGGTAAT-3'	Eurofins	OKS2184
PCR forward primer to generate insect cell expressed Mto2:<br>5'-GGGGTGATCAATGTCTGAACATAATTACCAG-3'	Eurofins	OKS2185
PCR reverse primer to generate insect cell expressed Mto2 and 6xHis-Mto2:<br>5'-GGTCGTCGACTTACTAGGGGGAAGGAGTGTCTTG-3'	Eurofins	OKS2186
PCR forward primer to generate insect cell expressed 6xHis-Mto2:<br>5'-GGGGTGATCAATGCATCATCATCATCATCACGAAAATCTTTACTTTCAGGGTTCTGAACATAATTACCAGTCT-3'	Eurofins	OKS2320
PCR forward primer to generate bacterial GST-Mzt-6xHis with Gateway cloning:<br>5'-GGGGACAAGTTTGTACAAAAAAGCAGGCTGGATCCGGATGGAGAACCTCTACTTCCAAGGTATGAGCGAAAGCACCAAAGAAACCATTGA-3'	Eurofins	OKS2874
PCR reverse primer to generate bacterial GST-Mzt-6xHis with Gateway cloning:<br>5'-GGGGACCACTTTGTACAAGAAAGCTGGGTCCTGCAGGGCGCCGGGCCCCTGGAACAGAACTTCCAGGCTCGGTTCGGTATCAACGGTCTG-3'	Eurofins	OKS2875
PCR forward primer to generate bacterial Mzt-6xHis with Gateway cloning:<br>5'-GGGGACAAGTTTGTACAAAAAAGCAGGCTGGATCCGGATGAGCGAAAGCACCAAAGAAACCATTGAAGT-3'	Eurofins	OKS2876
PCR reverse primer to generate bacterial Mzt-6xHis with Gateway cloning:<br>5'-GGGGACCACTTTGTACAAGAAAGCTGGGTCCTGCAGGGCGCCGGGCCCCTGGAACAGAACTTCCAGGCTCGGTTCGGTATCAACGGTCTG-3'	Eurofins	OKS2875
PCR forward primer 1 for N-terminal Strep-tag of Mto1[bonsai]:<br>5'-gggCTCGAGatgGGAAGCGCTTGGAGCCACCCGCAGTTCGAAAAAGGTGGAGGTTCTGGCGGTGGATCGGGAGGTTCAGCGTGGAGCCACCC-3'	Eurofins	OKS2883
PCR forward primer 2 for N-terminal Strep-tag of Mto1[bonsai]:<br>5'-GGGAGGTTCAGCGTGGAGCCACCCGCAGTTCGAGAAACTGGAAGTTCTGTTCCAGGGGCCCACTACTGATGATGAACAGCTTTCTCCCGTA-3'	Eurofins	OKS2884
PCR reverse primer for N-terminal Strep-tag of Mto1[bonsai]:<br>5'-ACCGCATGCTATGCATTTATTACTGGAGT-3'	Eurofins	OKS2978
PCR forward primer for tagging Mzt1 with nmt81:3xFLAG:<br>5'-CCTATTCCAGTAAACTTAACATTACCAACTTGTTACGAAGTATATATAATTAGACTAGTTTATTATAAAGTATCGAGTTTTGAATTCGAGCTCGTTTAAAC-3'	Eurofins	OKS2671
PCR reverse primer for tagging Mzt1 with nmt81:3xFLAG:<br>5'-CGTTGTTTTATCAAGTTCAGTTCCAAGCAATGTTCCTATTTCGTATAAAACTTCTATTGTCTCTTTGGTAGATTCAGACATTCCGCCTCCTTTATCATCATCGTCCTTATAG-3'	Eurofins	OKS2672
PCR forward primer for tagging Mto1[bonsai] with mECitrine:<br>5'-TGATGAGAATGGAGCAACAATGGCGGGAAGATGTTGACCAACTCCAGGAATATGTCGAAGAGATTACCCAAGAACTCCAGCGGATCCCCGGGTTAATTAA-3'	Eurofins	OKS2105
PCR reverse primer for tagging Mto1[bonsai] with mECitrine:<br>5'-TATAAATCTTGGAATTAGCTTATTTTGTCAATTAAATTCTCCAACTAAGTAGATATGTACTGGTGATTAAGAAAAGCTATGAATTCGAGCTCGTTTAAAC-3'	Eurofins	OKS2116

Table S1. Oligonucleotides used in this work. Related to STAR Methods and Key Resources Table.

Supplemental References

S1. Borek, W.E., Groocock, L.M., Samejima, I., Zou, J., de Lima Alves, F., Rappsilber, J., and Sawin, K.E. (2015). Mto2 multisite phosphorylation inactivates non-spindle microtubule nucleation complexes during mitosis. Nat Commun *6*, 7929.

S2. Dhani, D.K., Goult, B.T., George, G.M., Rogerson, D.T., Bitton, D.A., Miller, C.J., Schwabe, J.W., and Tanaka, K. (2013). Mzt1/Tam4, a fission yeast MOZART1 homologue, is an essential component of the gamma-tubulin complex and directly interacts with GCP3(Alp6). Mol Biol Cell *24*, 3337-3349.

S3. Masuda, H., Mori, R., Yukawa, M., and Toda, T. (2013). Fission yeast MOZART1/Mzt1 is an essential gamma-tubulin complex component required for complex recruitment to the microtubule organizing center, but not its assembly. Mol Biol Cell *24*, 2894-2906.

S4. Flory, M.R., Morphew, M., Joseph, J.D., Means, A.R., and Davis, T.N. (2002). Pcp1p, an Spc110p-related calmodulin target at the centrosome of the fission yeast Schizosaccharomyces pombe. Cell Growth Differ *13*, 47-58.

S5. Fong, C.S., Sato, M., and Toda, T. (2010). Fission yeast Pcp1 links polo kinase-mediated mitotic entry to gamma-tubulin-dependent spindle formation. EMBO J *29*, 120-130.

S6. Sawin, K.E., Lourenco, P.C., and Snaith, H.A. (2004). Microtubule nucleation at non-spindle pole body microtubule-organizing centers requires fission yeast centrosomin-related protein mod20p. Curr Biol *14*, 763-775.

S7. Venkatram, S., Tasto, J.J., Feoktistova, A., Jennings, J.L., Link, A.J., and Gould, K.L. (2004). Identification and characterization of two novel proteins affecting fission yeast gamma-tubulin complex function. Mol Biol Cell *15*, 2287-2301.
